# An optimization-embedded simulation approach for quantifying the operational impacts of right-sizing prenatal care

**DOI:** 10.1007/s10729-026-09782-6

**Published:** 2026-07-27

**Authors:** Leena Ghrayeb, Amy Cohn, Ruiwei Jiang, Alex Peahl

**Affiliations:** 1 Department of Industrial and Operations Engineering, University of Michigan, Ann Arbor, 48109 MI USA; 2 Department of Obstetrics and Gynecology, University of Michigan, Ann Arbor, 48109 MI USA

**Keywords:** Prenatal care, Discrete event simulation, Mixed-integer linear programming, Scheduling

## Abstract

The United States spends approximately $111 billion annually on maternity care but has the worst maternal mortality rate among peer high-income nations. Recent studies suggest that outdated prenatal care guidelines may be a cause for this. In response to the growing need for modernized prenatal care standards, national prenatal care stakeholders are moving away from the traditional “one-size-fits-all” prenatal care pathways and have proposed new “tailored” appointment pathways. To study the operational impacts of adopting this new paradigm, we propose a discrete-event simulation model, which captures patient-related heterogeneity, with a mixed-integer linear programming model embedded within it, to schedule patients on a weekly basis. The objectives are to minimize patient delays, patient rescheduling, and overbooking. We use this model to quantify the operational impacts of adopting the new tailored care paradigm and to draw insights about varying scheduling policies. We apply our model to a case study of a single prenatal care clinic within a large academic health center. Our results suggest that tailoring care significantly reduces delays, rescheduling, and overbooking. This additional flexibility may allow a clinic to accommodate more patients, or to better adapt to social risk factors and/or unexpected complications that require additional care. We also find that scheduling appointments one at a time, rather than by trimester or the entirety of the pathway, yields schedules with minimal delays and overbooking.

## Highlights

- We develop a discrete-event simulation model with a mixed-integer linear programming model embedded within it to schedule prenatal care appointments for patients with varying attributes.
- We show that the reductions in patient delays, rescheduling, and overbooking that result from adopting a tailored prenatal care paradigm are significant, reducing the operational burden on clinics.
- We observe that scheduling prenatal care appointments one-at-a-time yields a schedule with the least amount of delay, rescheduling, and overbooking.

## Introduction

**Fig. 1:**
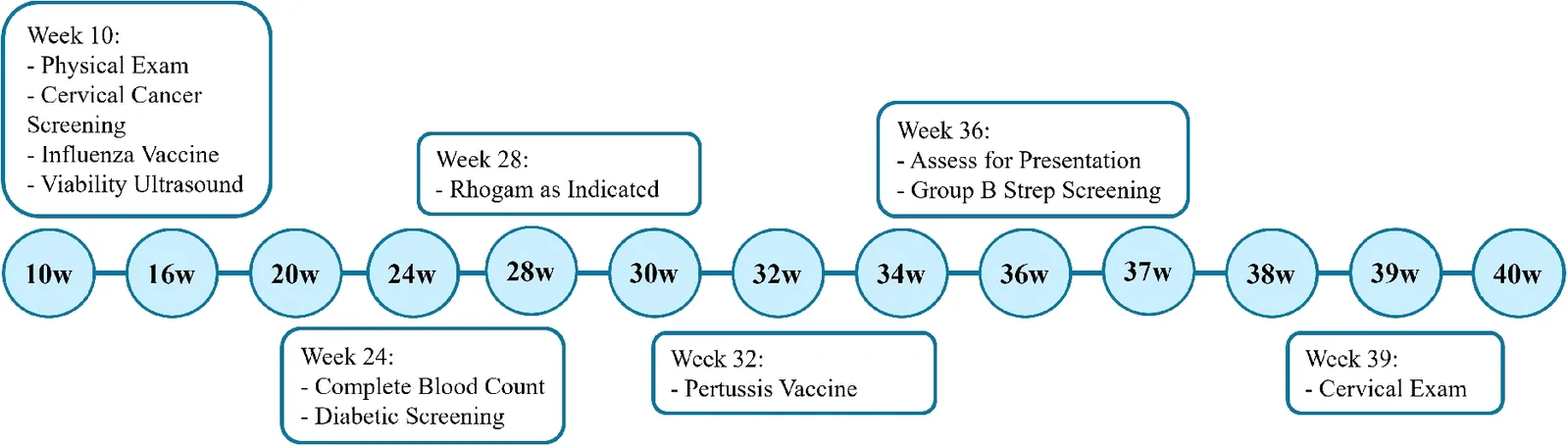
Gestational ages at each appointment in the status-quo prenatal care pathway

Despite the fact that the United States spends approximately $111 billion annually on maternity care, it has the worst maternal mortality rate among peer high-income nations [15]. Adverse outcomes disproportionately affect patients of color – the maternal mortality rate for non-Hispanic Black patients is 2.5 times the rate for non-Hispanic White patients and 3.1 times the rate for Hispanic patients [17]. Receiving prenatal care early in the pregnancy (i.e., within the first trimester, or first three months) is important to increase the chances of a healthy pregnancy, but evidence shows that patients of color are disproportionately impacted on this front too – only 70.5% of Hispanic patients and 67.6% of non-Hispanic Black patients received prenatal care during their first trimester, compared to 82.6% of non-Hispanic White patients [23]. Those living on low incomes or in rural areas are also more likely to face access issues. Generally, significant inequities exist in current prenatal care delivery, which has highlighted the need for a paradigm restructuring.

Prenatal care delivers evidence-based care throughout the course of a patient’s pregnancy, including screenings, vaccinations, and laboratory tests. A prenatal care *pathway* is defined as the sequence of appointments that a patient will receive during pregnancy, where each appointment occurs at a specific point in the pregnancy. Recent studies show that while the services delivered during routine appointments improve outcomes, the structure of the prenatal care pathways may not. The current recommendation of 12–14 in-person appointments over the course of the pregnancy was published in 1930. Despite significant medical and technological advancements since then, it has remained unchanged (Fig. 1). In fact, recent observational studies show that for medically low-risk patients, more than ten prenatal care visits add additional interventions in pregnancy with no improvement in outcomes – low-risk patients who received more than 10 appointments were more likely to have a cesarean-section delivery or induction of labor than those who did not [5]. Additionally, peer high-income nations with lower maternal mortality rates recommend fewer appointments than the U.S. for medically low-risk patients [25]. This implies that current guidelines are both inconvenient for medically low-risk patients and insufficient for patients who may need higher flexibility during their care. For clinics, these unnecessary appointments limit appointment availability, ultimately contributing to poor access.

In addition to the growing need for modernization of guidelines, the 2020 COVID-19 pandemic was a catalyst for rapid changes in prenatal care delivery. The American College of Obstetricians and Gynecologists (ACOG), in collaboration with the University of Michigan, convened a panel of national prenatal care stakeholders and experts including patients and public members to determine a new set of guidelines. The resulting paradigm, called the Michigan Plan for Appropriate Tailored Healthcare in Pregnancy (MiPATH), incorporates targeted visit schedules for medically average/low-risk patients, as well as the possibility of telehealth visits for all but four key appointments, during which screenings, vaccinations, and physical exams occur [9]. For simplicity, we call these appointments “anchor" appointments. MiPATH shifts from the status-quo “one-size-fits-all" model of care and instead tailors care to patients’ unique medical needs. While social support resources and addressing non-medical needs are an integral part of prenatal care, the introduction of a *medically* right-sized approach aims to ensure that patients’ medical needs are met. This approach also removes unnecessary appointments from their schedules, allowing clinics to be more flexible to unanticipated changes in patient care plans and to accommodate new and/or high-risk patients more easily.

As this new model of care is implemented, national prenatal care stakeholders, including health system leaders, clinicians, and policy-makers, are interested in quantifying its impacts on clinic scheduling and capacity utilization, from both the patient and provider perspectives. From the patient perspective, a congested clinic schedule may mean very little flexibility in the days that they can be scheduled for an appointment, causing a patient to either be delayed in their care, or even to skip an appointment [18]. For clinics, limited capacity and a growing demand for prenatal care appointments lead to overbooking, which requires additional work hours from providers and staff, as well as other possible additional costs. For prenatal care providers, increasing demand and heavy workloads have contributed significantly to burnout and job dissatisfaction [32]. It is hypothesized that by tailoring care to patients’ specific medical needs, patients’ ability to access care when they need it will increase, while reducing the burden on providers.

In addition to tailoring care, there is a complementary but separate interest among stakeholders to consider alternative scheduling policies. We consider two main questions:

*Given that a patient’s prenatal care pathway is known as soon as they initiate care, how many appointments should be scheduled at a time?* In the current state, patients have varying preferences regarding the scheduling of their pathways. Some patients prefer to have all of their prenatal care appointments scheduled as soon as they initiate care, as this allows them to plan for childcare, time off work, or other accommodations, but this may cause clinics to preemptively reserve appointment slots that may not actually be needed. Others desire more flexibility and would rather schedule their appointments by trimester, or even one at a time, but these policies also imply limited knowledge about the timing of future appointments past a certain time horizon. From a provider perspective, scheduling appointments by trimester or one at a time allows for greater flexibility and adaptability to a patient’s dynamic health state, as a patient’s appointment timing or frequency may change due to unanticipated symptoms during pregnancy.

*How should a clinic schedule a patient if it does not have capacity to accommodate an appointment in the required week?* Clinics may choose to overbook, meaning booking appointments even when there are no appointment slots available, to ensure that patients receive care when they need it. However, this is burdensome for providers and results in a congested schedule and extra work time. Alternatively, clinics may choose to delay a patient’s appointment to a later week that does have available appointment slots, especially if the appointment is not an anchor appointment, but this may delay the diagnosis or management of new symptoms or conditions (e.g. high blood pressure). Similarly, if the appointment is an anchor appointment, the clinic may choose to reschedule another patient’s already scheduled non-anchor appointment to a later week so that the anchor appointment can be scheduled in its place, on-time. However, rescheduling is inconvenient for patients who may have already taken time off of work or made childcare or transportation plans. It is important to note that in practice, clinics tend to exclusively overbook, but given the trade-offs that exist within all of the mentioned policies, we wish to understand the effects of adopting them.

Adopting a tailored paradigm leads to a reduced appointment volume in clinics, implying a reduction in delays and overbooking. Although this is intuitive, quantifying the magnitude and robustness of these reductions is an important aspect in understanding the operational impacts of these new policies. The primary objectives of this article are two-fold: 1) to propose and validate a data-driven model that quantifies the effects of adopting a “right-sized" prenatal care paradigm and 2) to draw insights about the best scheduling policies for prenatal care appointments.

To achieve these objectives, we propose an optimization-embedded simulation model, which consists of a mixed-integer linear program (MILP) to optimally schedule patients, with the objectives of minimizing the costs associated with patient delay, overbooking, and rescheduled appointments, embedded within a data-driven simulation model that captures patient-related heterogeneity. We use this model to provide insights on proposed policies that are under consideration for implementation in clinics. We test different cost schemes that capture varying provider preferences for overbooking and delaying appointments and draw insights about best practices for scheduling prenatal care appointments.

This article is organized as follows: in Section 2, we provide a review of relevant literature. In Section 3, we describe our problem and key assumptions. In Section 4, we outline our methodology, including data analysis and our proposed optimization-embedded simulation model. In Section 5, we present our experimental results and discuss them in Section 6. In Section 7, we summarize insights gained from our experiments and conclude with future research directions.

## Literature review

Generally, there have been very few studies that focus on the scheduling of prenatal care appointments specifically. Prenatal care is provided in obstetrics and gynecology (Ob/Gyn) clinics, which provide care related to pregnancy and the female reproductive system. Prenatal care can also be delivered by family medicine doctors, nurse practitioners, and family medicine doctors in a variety of settings: primary care clinics, federally qualified health centers, and public health clinics. In this study, we focus on Ob/Gyn clinics. Lenin et al. [21] propose a simulation model to determine the optimal time between appointments, as well as number of nurses, to reduce patient wait time in an Ob/Gyn clinic. Anvaryazdi et al. [2] propose a two-stage stochastic programming model to generate a scheduling template for providers to schedule appointments and resources. Fun et al. [10] propose a simulation model to evaluate the operational impact of changing consultation times and patient arrival times. All of these studies focus on reducing patient wait times, include both obstetric and gynecologic patients in their models, and only consider single-appointment scheduling of appointments, not the entire prenatal care pathway.

From a methodological perspective, the most closely related problem to ours within the literature is that of outpatient chemotherapy appointment systems (OCAS). There have been two review papers summarizing works related to OCAS and both note that this is a fairly new application area that is quickly growing [13, 19]. Similar to prenatal care, chemotherapy follows a cyclic appointment pattern, where patients visit the clinic on specific treatment days which are separated by a fixed number of rest days [7]. It is usually administered in an outpatient setting. This scheduling problem is unique in that it contains two different patterns: multi-day patterns, which is the sequence of treatment days with rest days in between, and intra-day patterns, which is the sequence of nurse activities and lags in between on the day of treatment [7]. Further complicating intra-day patterns is the high variability in resource requirements for each patient, as well as many different hospital resources/personnel required [31]. Many articles consider either scheduling of multi-day or intra-day patterns and some consider both simultaneously [7]. Since our proposed problem assumes identical intra-day patterns for each appointment slot, we do not include literature that only addresses scheduling for intra-day patterns. Herein, we categorize relevant studies based on methodology.

### Mixed integer linear programming

Many studies utilize a MILP approach. Due to the complexity of the formulation, heuristics are commonly used to find good solutions in a reasonable computational time.

Turkcan et al [31] address the problem of scheduling patients for their chemotherapy treatment, taking into account scheduling the day of the treatment, as well as the time slot per day. They consider availability of resources (nurses/pharmacists and number of chairs/beds), with the possibility for overbooking, as well as patient acuity as environmental factors. They formulate this problem as a two-stage MILP problem and propose a rolling horizon algorithm to solve it. Similarly, Condotta and Shakhlevich [7] consider the problem of multi-day and intra-day scheduling for chemotherapy patients, but differ from Turkcan et al. [31] in that they do not treat intra-day patterns as contiguous blocks of time, but consider time-lags between different nurse activities. They also differ in the scheduling approach; while Turkcan et al. [31] performs scheduling at regular times, Condotta and Shakhlevich [7] perform scheduling dynamically as patients arrive. They propose a multilevel template schedule methodology, where the schedule is constructed in stages through solving MILPs. The first stage determines which day each patient will be scheduled, while the second stage determines which time slot in a day each patient will be scheduled. The objectives are to minimize patient delay and nurse workloads. Once this schedule is generated, it is adjusted as new patients arrive, also through solving a MILP.

Sadki et al. [27] propose a three-stage MILP approach for the multi-day problem to determine the consultation times of oncologists, assignment of patients to beds, and the number of patients consulted by interns. The objective is to best balance the bed capacity requirement over the planning horizon.

[20] consider the problem of scheduling chemotherapy appointments with the objectives of minimizing overtime, maximizing the number of patients scheduled, and minimizing variance in workload among nurses. They formulate this problem as a MILP and propose a metaheuristic algorithm to solve it. Their method generates an initial solution with a greedy algorithm and then improves the initial solution with Tabu Search.

Hooshangi-Tabrizi et al. [16] propose a flexible and adaptive integer programming-based procedure for online scheduling, which allows for adaptive appointment scheduling in the event of absent nurses, unexpected equipment breakdown, treatment regimen changes, or patient cancellations. Their first model takes into account the number of chairs and nurses available and aims to minimize the number of patients assigned to float nurses, the number of patients who do not receive their preferred time slot, nurse overtime, and the number of appointment requests that are directed to a buffer and not scheduled. The second model allows for rescheduling of already booked appointments and considers a maximum allowable number of time slots for which a treatment may be moved, the maximum percentage of appointments that can be moved, and the maximum amount of time by which the start time of a treatment may change. This procedure is triggered when there is an incoming list of patients, or when there is an unexpected event.

Tran et al. [30] model the multi-day scheduling problem using the hybrid flow shop model as a theoretical framework and propose a MILP, with the objective of minimizing the total completion time of a patient’s regimen. They also propose a genetic algorithm and compare its results against CPLEX.

### Markov decision process

Few researchers propose markov decision process (MDP) models. Sauré et al. [29] propose an MDP approach to schedule radiotherapy appointments, with the objectives of minimizing patient waiting time, overtime, and postponing booking decisions. Similarly, Gocgun and Puterman [12] employ an MDP approach for the problem of chemotherapy scheduling. Decisions are made at the end of each day over an infinite horizon, taking into account new patient arrivals daily. Decisions consist of either scheduling a new patient for their appointments, or diverting the decision for a future time. They consider constraints on daily resource capacity, maximum number of patients diverted, and overbooking. Unlike Sauré et al. [29], this study assigns zero cost for scheduling a patient within a given tolerance and a linear cost for either scheduling them early or late, along with a diversion cost. Like Sauré et al. [29], they implement a linear programming based approximate dynamic program to solve the problem and find that their methodology outperforms simple heuristics in cases where patients can be scheduled outside their tolerance limits.

### Simulation

Other researchers incorporate simulation into their solution approaches. Heshmat and Eltawil [14] consider the problem of both multi-day and intra-day scheduling of chemotherapy patients and propose a two-stage approach. In the first stage, an MILP is formulated to schedule patients to a day, taking into account resource constraints (drugs, pharmacists, chairs/beds, and nurses), with the objectives of minimizing the weighted sum of treatment delay and total completion time for all treatments. In the second stage, they develop a discrete event simulation model to determine the optimal daily schedule based on the daily sequence of tasks undertaken by the nurses and pharmacists.

Sadki et al. [26] address only multi-day scheduling of chemotherapy, taking into consideration bed capacity, number of physicians, and physician capacity. Their work is one of the few that considers oncologist consultation in the outpatient setting. They propose a two-stage model, in which simulation is used to generate weekly arrivals of patients, and patients are scheduled weekly using a MILP with objectives of best smoothing of the bedload of each week and minimizing overbooking.

Benzaid et al. [4] consider both multi-day and intra-day scheduling of chemotherapy patients, where the first stage is to assign patients to a day and a time and the second stage is to solve a staff planning problem. They propose a sequence of MILPs, with a simulation model to generate inputs.

Ghrayeb et al. [11] propose a discrete-event simulation to measure the reductions in patient delay and overbooking that result from adopting a tailored care policy, and answer questions related to the allocation of capacity (i.e., telehealth vs. in-person). This work provides the foundation for one component of our current work, namely the discrete-event simulation model. However, our study differs from [11] in a few key ways:

- The previous study’s objectives were to quantify the impacts (i.e., in overbooking and patient delay) of adopting a tailored prenatal care policy, and to study the best capacity allocation policies for prenatal care within a large academic health center. Our proposed study expands on these objectives by 1) including rescheduling in the objective and 2) considering questions related to scheduling policies related to prenatal care.
- Methodologically, the previous study focused on developing a discrete-event simulation model, which simulates patients with heterogeneous prenatal care pathways and schedules them for their appointments based on a first-come first-serve policy. Our current study expands on this model by embedding an MILP within this simulation model, allowing us to dynamically schedule patients over the course of the horizon.
- Finally, our proposed study incorporates an additional layer of patient complexity – low risk patients can switch to a high-risk pathway mid-pregnancy if certain complications arise. This brings our model(s) closer to real-world patient behavior, and allows us to draw more accurate conclusions about our policies.

### Stochastic programming

Few researchers implement stochastic programming methods. Alvarado and Ntaimo [1] propose three mean-risk stochastic integer programming models, called SIP-CHEMO, to solve the problem of scheduling chemotherapy patients to appointments and resources. They take into consideration the uncertainty of patient acuity, appointment duration, and nurse availability. The first model assigns patients to appointment days, specific chairs, and a nurse, with the objectives of minimizing deviation from a patient’s appointment pathway and filling time slots with the highest priority. The second model’s objective is to minimize scheduling conflicts for overtime, excess acuity, and appointment overlaps. The third model takes into account the expected value for excess over a target objective function value (e.g. maximum number of days the schedule can deviate from the recommended treatment date). They find that their approach significantly decreases patient waiting time and nurse overtime compared to deterministic scheduling algorithms.

Castaing et al. [6] and Demir et al. [8] both propose two-stage stochastic integer programs to determine patient appointment times and chair assignments in outpatient chemotherapy clinics, given uncertainty in infusion durations. However, Castaing et al. [6] assume that an initial schedule has already been created, whereas Demir et al. [8] create the initial schedules. Additionally, Castaing et al. [6] propose a model that must be solved for each nurse in the clinic, while Demir et al. [8] propose a model that takes into account multiple nurses. Both models address the problem of intra-day scheduling, so the application of stochastic programming techniques to multi-day scheduling for similar problems is still fairly sparse.

**Fig. 2:**
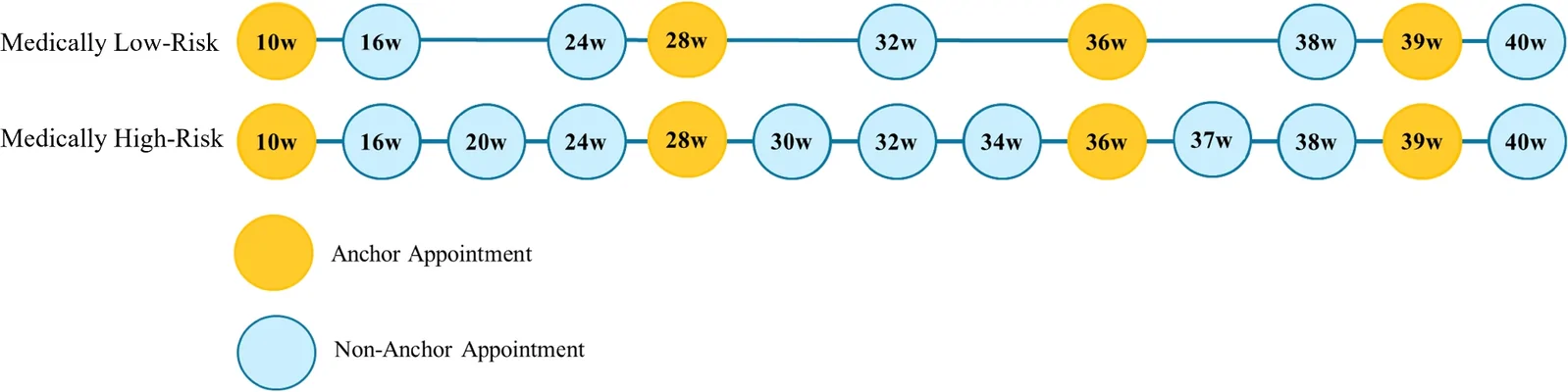
Gestational ages at each appointment in the tailored prenatal care pathways

### Contributions and impact

In the broader literature, to the best of our knowledge, this is the first work to apply optimization methods to the problem of multi-day scheduling of prenatal care appointments. While prenatal care scheduling is similar to radiotherapy and chemotherapy scheduling in that patients require a sequence of appointments, our prenatal care setting is unique in that certain prenatal care appointments have higher priority than others, allowing the possibility of delaying or rescheduling lower-priority appointments. Additionally, we propose the first model to consider minimizing patient delay, rescheduled appointments, and overbooking simultaneously.

This work also has significant implications for healthcare policy. As this new prenatal care paradigm is currently being adopted by 150 providers across 12 clinics in Michigan, with the strong potential for its adoption in clinics across the country, this research will directly inform decision-making [3].

## Problem description and assumptions

We consider one outpatient Ob/Gyn clinic, which has a limited number of appointment slots per week. The clinic’s capacity is split between obstetrics (focused on pregnancy, childbirth, and post-childbirth care) and gynecology (focused on care related to the female reproductive system). We assume that the clinic allocates 50% of its capacity to each. We only consider the problem of the weekly scheduling of appointments and do not consider intra-day scheduling. We also assume that patients are punctual, so we do not consider patient tardiness or no-shows.

Upon arrival to the system, patients are classified based on their medical risk factors and are assigned a pathway accor-dingly. Pathways are given by a sequence of weeks during which the patients should be seen by a prenatal care provider. During these appointments, patients receive maternal and fetal assessment (e.g. blood pressure checks, vaccines, screenings, etc.) as well as anticipatory guidance and counseling. In the MiPATH model of care, there are four anchor appointments, during which vaccinations, physical exams, imaging, and laboratory tests are completed. Anchor appointments can only be delayed by one week if needed. In the “one-size-fits-all" model, all patients follow a 13-appointment in-person pathway, while in the MiPATH model, medically low-risk patients follow a 9-appointment pathway (Fig. 2).

Additionally, patients initiate care at varying gestational ages (i.e., week in their pregnancy) and give birth at varying gestational ages, so the number of appointments they have during their pregnancies varies. Low-risk patients can also switch to a high-risk pathway mid-pregnancy if they are diagnosed with a condition that requires additional care. While there are numerous conditions that may cause a patient to require additional appointments beyond their pathway, clinical collaborators identified the two most common conditions: hypertensive disorders (gestational hypertension or preeclampsia) and gestational diabetes. Both of these conditions can have significant adverse effects on the patient and the fetus, requiring additional monitoring and specific treatment plans during the pregnancy [22]. In our model, we assume that if a low-risk patient is diagnosed with either a hypertensive disorder or gestational diabetes mid-pregnancy, they switch to the high-risk pathway at the week of diagnosis. If a high-risk patient is diagnosed, they remain on their original care pathway. In terms of scheduling, this means that the model now has to schedule additional appointments (up to four more appointments) for any low-risk patient that is diagnosed. At the clinic level, this added randomness in demand for appointments makes planning much more complex.

We assume that at the end of each week, the clinic schedules appointments for all patients who have arrived in that week, and existing patient appointments have the potential to be rescheduled, depending on the clinic’s policy. We consider the following three scheduling protocols:

1. All up-front policy: The clinic schedules all appointments for the pregnancy when the patient initiates care. The patient and the clinic know when the patient will have appointments during the entirety of their pregnancy.
2. Trimester policy: Pregnancies are split into three segments of approximately three months, called trimesters. In this policy, the clinic schedules only the current trimester’s appointments. (e.g., if the patient arrives during the first trimester, the clinic schedules all of the first trimester’s appointments. Once they complete the first trimester, the clinic schedules all of the second trimester’s appointments, and so on.)
3. Single appointment policy: The clinic schedules one appointment at a time. When a patient completes an appointment, they are scheduled for their next one.

Scheduling all prenatal care appointments up-front when a patient initiates care would allow a patient to plan for childcare, transportation, or other logistics far in advance of an appointment, but may cause clinics to prematurely make poor scheduling decisions based on the expectation that an appointment will be needed in a certain week. For example, low-risk patients may switch to high-risk pathway mid-pregnancy, which would require extra appointment slots beyond those that have been scheduled. Additionally, a patient may give birth before the end of their care pathway, causing any unused appointments to be canceled, meaning the clinic had preemptively reserved an appointment slot(s) for that patient that could have been used for another patient. Scheduling appointments by trimester is similar in that it offers patients advance knowledge of future appointments, albeit for a shorter window into the future, while allowing clinics slightly higher flexibility to adapt to changes in patients’ needs. Scheduling appointments one at a time offers the highest flexibility for clinics, but does not offer patients the benefit of future knowledge of their appointment dates.

If an appointment needs to be scheduled in a week where there is no capacity left, the clinic can take one of the following actions:

1. Delay the appointment within a certain delay threshold. Delaying appointments is unfavorable from a clinical perspective, as appointments should be scheduled as close as possible to their target weeks in the pathway to ensure the best health outcomes. However, delays may be needed due to insufficient capacity. The negative effects of delaying appointments are minimized by adhering to defined delay thresholds, based on the type of appointment. For example, appointments that have key screenings or vaccinations can only be delayed by one week, while other appointments may be delayed more. While there have not been any studies that rigorously tie delaying individual prenatal care appointments to health outcomes, it is clinically well-established that prenatal care is an integral part of the timely management of complications during pregnancy [24]. This is especially true of key appointments during which essential screenings occur, like the screening for gestational diabetes. Additionally, delayed initiation of prenatal care (i.e., patients starting their prenatal care after the first trimester), is linked to a significant increase in the risk for adverse outcomes like pre-term birth, cesarean section, and gestational diabetes [28].
2. Reschedule an existing appointment from a previously scheduled patient to a later week. For example, if the clinic needs to schedule an anchor appointment, but there is no capacity in its target week, an existing non-anchor appointment for another patient may be rescheduled and delayed to a later week to accommodate it. From a clinical perspective, this is a trade-off that allows key appointments to be scheduled when they need to be, while delaying less important appointments. Due to the sequential nature of prenatal care appointments, it is not clinically favorable to delay any appointment, but the negative consequences of delaying an anchor appointment are much more significant than those of delaying a non-anchor appointment. Note that it is also possible for an initially delayed appointment to be rescheduled to an earlier week if capacity opens up in its target week, due to another patient delivering before the end of their pathway. Rescheduling appointments is also inconvenient for patients, who may have already made plans to attend their appointment in its originally scheduled week. To minimize patient inconvenience, appointments that have already been scheduled to occur within the next month are not eligible to be rescheduled.
3. Overbook the appointment. Overbooking appointments allows patients to receive their care on-time, but results in extra costs for the clinic, including the time of providers and staff and operational costs.

To determine the quality of the schedule, three metrics are defined.

1. Patient Delay: the number of weeks delayed an appointment is from its target week.
2. Patient Rescheduling: the number of times a patient has an appointment rescheduled.
3. Overbooking: the number of appointments that are overbooked.

These metrics are included in the objective of the optimization model embedded within the simulation model. Note that we only capture the “realized" metrics, meaning delay, overbooking, and rescheduling are only counted if and when an appointment actually occurs. For example, if an appointment is deleted from the schedule due to a patient delivering before the end of their pathway, that appointment’s associated metrics are not included in the final results. It is also possible that an appointment is rescheduled multiple times before it actually occurs; in this case, delay and overbooking are only counted for that appointment in the week that it occurs, and rescheduling is counted for the entire time that the appointment/patient has been in the schedule.

**Fig. 3:**
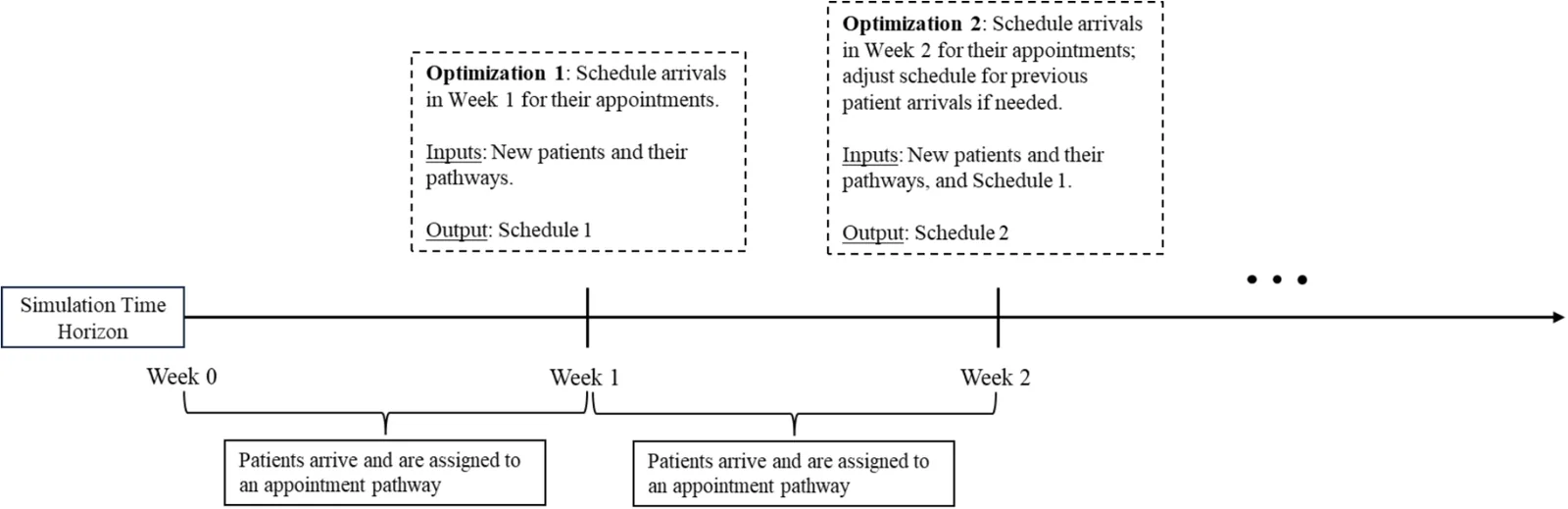
Optimization Embedded Simulation Overview

## Methodology

The proposed methodology consists of two components. The first component is a discrete-event simulation model, which simulates patient arrivals, their classifications based on medical risk factors, gestational age, corresponding appointment pathways, and conditions that could potentially develop during pregnancy. The second component is a mixed-integer linear programming model (MILP), which is used to find the optimal schedule for patients in the system. The optimization model is embedded within the simulation model, such that at the end of every week in the simulation, all new arrivals for that week are scheduled for their appointments using the optimization model and existing patients are rescheduled if needed. We chose an MILP to model our policies rather than directly implementing a simple policy within the simulation model (e.g., a first-come first-serve approach) due to the complexity of our policies and the possible tradeoffs, as well as the ability to easily capture varying provider preferences for the different actions (i.e., in the MILP, we can easily vary the costs within the objective function to generate a Pareto front, which would be difficult to capture with a simple policy). This optimization of the schedule occurs every week, for the entire time horizon of the simulation. Figure 3 shows a high level overview of this framework. During the first week of the simulation horizon, the simulation model generates patient arrivals, along with their attributes and pathways. At the end of the week, the optimization model takes the information generated by the simulation model as inputs and generates a schedule for the next 60 weeks in the horizon. During the next week, the simulation generates new patient arrivals. The optimization model is then re-run, with the new patient arrivals and their attributes as inputs, as well as the schedule generated during the first week (which is adjusted such that it contains the next 59 weeks in the horizon, given that the first week has already passed). The optimization model outputs a new schedule for the next 60 weeks (or for the rest of the weeks in the simulation horizon), which includes the patients/appointments scheduled after the first week and the new patients/appointments. This process continues until the end of the simulation horizon. While in our inputs, it is impossible for an appointment to be scheduled beyond 40 weeks from a patient’s arrival, we choose 60 weeks for our optimization horizon to allow for future flexibility – we may choose to incorporate patients whose pregnancies exceed 40 weeks (typically up to 42 weeks), scheduling of follow-up appointments after delivery, or appointments related to social support, which could extend beyond the pregnancy’s 40-week horizon. This optimization horizon can be easily adjusted for users’ needs.

### Discrete event simulation

Given the assumptions and problem description given in Section 3, a discrete event simulation model was developed in C++ to model patient arrivals and their attributes. This model uses inputs based on historical data to generate a number of patient arrivals each week, along with their medical risk level, gestational age at arrival, and gestational age at delivery. For low-risk patients, the model also generates whether the patient will be diagnosed with a hypertensive disorder or gestational diabetes and the gestational age that they are diagnosed and switch to high-risk pathway. At the end of the week that the low-risk patient is diagnosed, the patient switches to the high-risk pathway. Their already scheduled appointments are retained, and the model schedules all additional appointments they may need beyond their original low-risk pathway. Note that when scheduling decisions are made for each patient, the model does not use any future knowledge about a patient’s changing risk level or delivery week to schedule appointments. For example, if we are testing the schedule appointments all up-front policy, all patients are scheduled for their entire prenatal care pathway (i.e., through week 40 of the pregnancy) and once the simulation reaches the week in which they give birth, unused appointments are canceled.

**Table 1:** Medical Factors Used for Patient Classification

Medical Factors
Hypothyroidism
Previous C-Section
Pre-Existing Diabetes
Chronic Hypertension
Asthma
Chronic Hepatitis
Connective Tissue/Autoimmune Disorders
Hyperthyroidism
Chronic Kidney Disease
Chronic Ischemic Heart Disease
Congenital Heart Disease
Cardiac Valvular Disease
Cystic Fibrosis

**Table 2:** Simulation Input Parameters

Parameter	Distribution/Value	Source
Arrival Rate (# of high-risk and low-risk patients/week)	Empirical	EHR Data
Gestational Age at Arrival	Empirical	EHR Data
Gestational Age at Delivery	Empirical	EHR Data
Delay Threshold (maximum number of weeks a non-priority appointment can be delayed in each trimester; priority appointments can only be delayed by one week –[Trimester 1, Trimester 2, Trimester 3])	[3,2,1]	Expert/Health System
Capacity (# of obstetrics appointment slots available at the clinic/week)	156	Expert/Health System
Overbooking Limit (# overbooked appointments allowed at the clinic/week)	16	Expert/Health System

To derive inputs for the simulation model, we analyzed a year-long data set from the health system’s electronic health record (EHR). This data set included all patients who gave birth at the health system from March 1st, 2021–March 31st 2022 and received routine prenatal care at any of the system’s ambulatory clinics. 4,992 patients were included. For each patient, we obtained information regarding their pre-existing conditions (e.g. history of high blood pressure). In collaboration with a group of prenatal care stakeholders, we identified a list of conditions that would likely cause a patient to follow a care pathway with a higher frequency of appointments (Table 1). Note that while this table provides conditions that could potentially lead a patient to be classified as medically high-risk, patient medical-risk classification is highly dependent on a patient’s specific needs. For example, while one provider may classify a patient that has received a c-section in the past as medically high-risk, another may decide that their patient’s health can be safely managed with a low-risk pathway. We found it difficult to identify this decision-making process in the data, as our data did not include a field for patient-risk. Instead, we erred on the side of caution – if a patient in our data set had any of the pre-existing conditions shown in Table 1, we classified them as medically high-risk. Based on these insights, of the 4,992 patients in the data set, 3,113 (62%) were classified as medically high-risk and 1,879 (38%) were classified as medically low-risk. This is likely an over-estimation of the real-world proportion of high-risk patients, implying that using these inputs within our model will provide a conservative estimate for the improvements in the metrics after the implementation of the tailored care pathways.

The data set also included appointment dates for each patient, the date of delivery, and the gestational age at delivery. Using these fields, we calculated arrival rates (number of patients/week) for each patient type, the distribution of gestational age at arrival for each patient type, and the distribution of gestational age at delivery for each patient type. Clinic-related data was obtained from the health system directly. Table 2 provides a summary of the input parameters used in the simulation model, as well as their sources. For parameters modeled by empirical distributions, the inverse transform method was used for sampling.

### MILP formulation

The following is the formulation for the MILP, used to schedule/reschedule patient appointments, which is embedded within the simulation model. This formulation is solved for each week in the simulation time horizon.

**Sets**:

$\{p \in P\}$: Set of all patients, including both existing patients and new arrivals.

$\{n \in N\}$: Set of all new patients (N⊂P).

$\{v \in V^p\}$: For each patient, the pathway appointments still left in their treatment relative to the current week and the scheduling policy. For example, in the schedule all up-front policy, this set includes all appointments left in their pathway relative to the current week for the rest of their pregnancy, whereas for the schedule by trimester policy this set includes all appointments left in the current trimester relative to the current week. This set does not include appointments that have already occurred.

$\{w \in W\}$: Set of all weeks in the planning horizon.

$\{s \in S\}$: Set of all stages (i.e., weekly).

**Decision Variables (for Stage** *s***)**:

$X_{pvws}$: Binary variable; 1 if patient *p* has pathway appointment *v* scheduled in week *w*, 0 otherwise.

$O_{ws}$: Total number of appointments overbooked in week *w*.

$Y_{pvs}$: Binary variable; 1 if patient *p* has pathway appointment *v* moved/rescheduled, 0 otherwise.

$T_{pvs}$: Total tardiness (weeks of delay) per patient *p*, per appointment *v*.

**Parameters**:

$c^T_v$: The cost per unit of tardiness (week) for pathway appointment *v*.

$c^O$: The cost for overbooking an appointment.

$c^Y_v$: The cost for rescheduling pathway appointment *v*.

*C*: Number of appointment slots available per week.

*F*: First week in the rescheduling window (i.e., appointments that have been scheduled to occur before week *F* cannot be rescheduled).

*L*: Maximum number of appointments overbooked per week.

$G_{pv}$: The target week that pathway appointment *v* should be scheduled for patient *p*.

$U_{pv}$: The maximum number of weeks that appointment *v* can be delayed for patient *p*.

$Z_{pvw}$: Schedule from the previous stage (only includes existing patients); 1 if patient *p* was scheduled for pathway appointment *v* in week *w*, 0 otherwise.

*R*: The maximum number of times a patient can be rescheduled.

$A_p$: The total number of times appointments have been rescheduled per patient; cumulative, after each stage $A_p$ is incremented by $Y_{pvs}$.

**Model (for any given stage** *s***)**:

Minimize

1 $$\begin{aligned} \sum \limits _{p \in P} \sum \limits _{v \in V^p}c^T_v T_{pvs} \ + \sum \limits _{w \in W} c^O O_{ws} + \sum \limits _{p \in P} \sum \limits _{v \in V^p}c^Y_v Y_{pvs} \end{aligned}$$

Subject to:

2 $$\begin{aligned}&\sum _{w \in W} X_{pvws} = 1, \ \forall \ p \in P, v \in V^p&\end{aligned}$$

3 $$\begin{aligned}&X_{pvws} = 0, \ \forall \ p \in P, v \in V^p, w \le G_{pv} - 1&\end{aligned}$$

4 $$\begin{aligned}&X_{pvws} = 0 \ \ \ \ \forall \ p \in P, v \in V^p, w > G_{pv} + U_{pv}&\end{aligned}$$

5 $$\begin{aligned} X_{pvws}&\le \sum _{u = 1}^{w-1} X_{pius},\nonumber \\&\forall \ p \in P, v \in V^p, i < v, w \in W \setminus \{1\} \end{aligned}$$

6 $$\begin{aligned}&O_{ws} \ge \sum _{p \in P} \sum _{v \in V^p} X_{pvws} - C, \ \forall w \in W&\end{aligned}$$

7 $$\begin{aligned}&O_{ws} \le L, \ \forall w \in W&\end{aligned}$$

8 $$\begin{aligned} Y_{pvs} \ge X_{pvws} -&Z_{pvw},\nonumber \\ \forall&\ p\in P \setminus N, v \in V^p, w \in W \end{aligned}$$

9 $$\begin{aligned}&\sum _{v \in V^p} Y_{pvs} + A_p \le R, \ \forall \ p \in P \setminus N&\end{aligned}$$

10 $$\begin{aligned} X&_{pvws} = Z_{pvw}, \nonumber \\&\forall \ p \in P \setminus N, v \in V^p, w < F: Z_{pvw} = 1 \end{aligned}$$

11 $$\begin{aligned}&T_{pvs} = \sum _{w \in W} (w - G_{pv})^+ X_{pvws}, \ \forall \ p \in P, v \in V^p&\end{aligned}$$

12 $$\begin{aligned}&X_{pvws} \in \{0,1\}, \ T_{pvs}, O_{ws} \ge 0&\end{aligned}$$

The objective (1) is to minimize the total cost of patient tardiness, overbooking appointments, and rescheduling appointments. Constraints (2) define that all appointments are scheduled within the planning horizon. Constraints (3) define that appointments cannot be scheduled before their target weeks. Constraints (4) define that an appointment cannot be scheduled past its defined latest week. Constraints (5) define that appointments must be scheduled in sequential order based on the appointment pathway. Constraints (6) define that the number of appointments overbooked in a given week is at least the difference between the number of appointments scheduled in that week and the standard capacity (excluding capacity set aside for overbooking) in that week. Constraint (7) define that the number of appointments overbooked per week cannot exceed the number of appointment slots available for overbooking (i.e., the overbooking limit). Constraints (8) define that an appointment is rescheduled if the current scheduled week differs from the scheduled week from the previous stage. Constraints (9) define that a patient can only be rescheduled a certain number of times over the course of their pregnancy. Constraints (10) define that appointments that have already been scheduled within a certain time window past the current week cannot be moved/rescheduled. Constraints (11) define that the tardiness per patient, per appointment, is the difference between the week scheduled and the goal week for that appointment. Constraints (12) give the binary and non-negativity conditions on the decision variables.

**Fig. 4:**
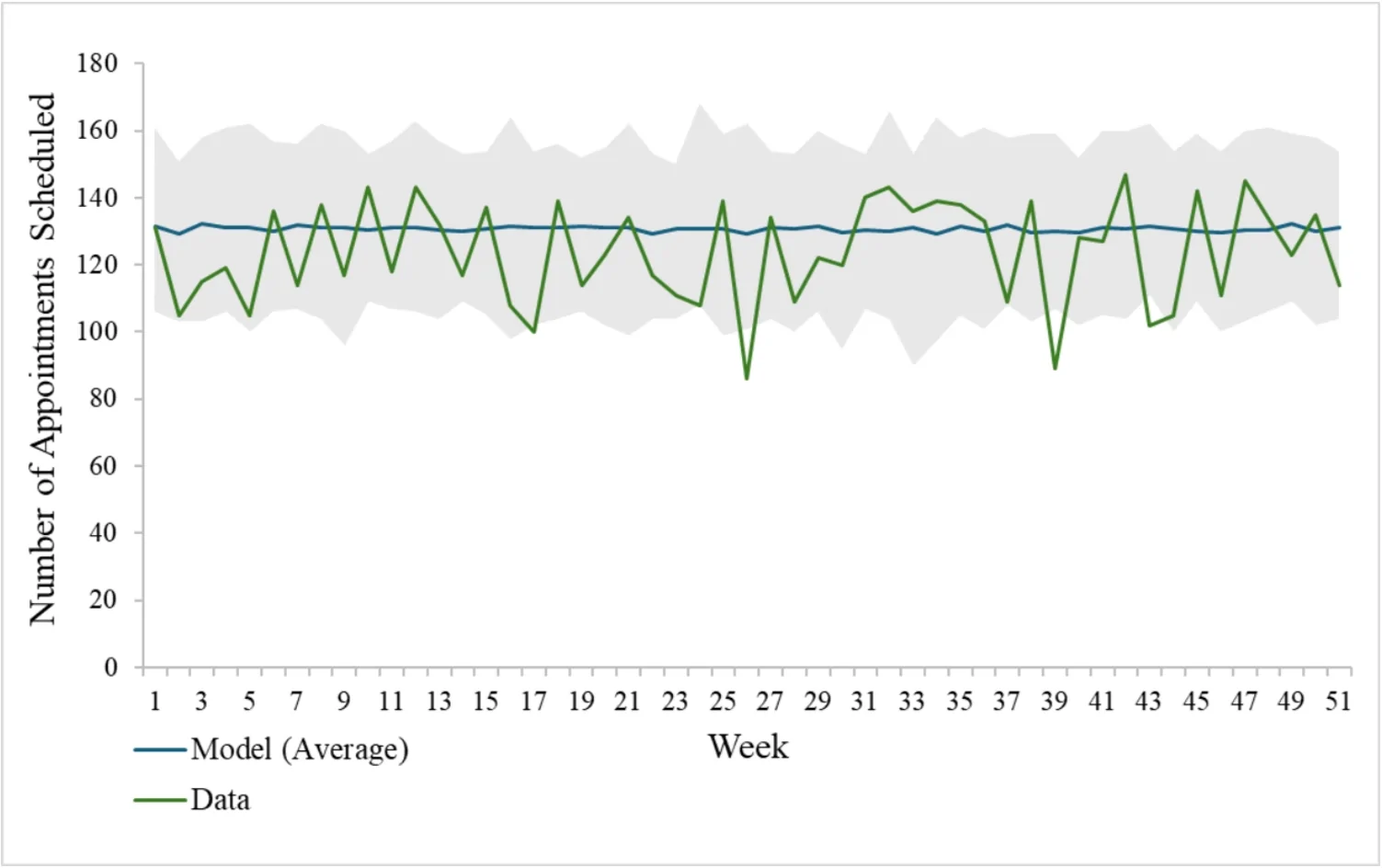
A comparison of the number of appointments scheduled per week in the model and historical data (the shaded region indicates the range of values obtained from 170 replications of the model)

This model may become infeasible if the demand for appointments exceeds the clinic’s capacity, including its overbooking capacity. Therefore, we modify this formulation to allow appointments to be scheduled even if there are no available appointment slots, with some high penalty cost. We define $I_{ws}$ to be the total number of appointments booked beyond the clinic’s overbooking capacity per week *w*. We also define *M* to be some very high cost (e.g., 1000). We then modify the objective  (1) and constraints  (6) as follows:

13 $$\begin{aligned} \sum \limits _{p \in P} \sum \limits _{v \in V^p}c^T_v T_{pvs} \ +&\sum \limits _{w \in W} c^O O_{ws} + \sum \limits _{w \in W} M I_{ws}\nonumber \\&\quad + \sum \limits _{p \in P} \sum \limits _{v \in V^p}c^Y_v Y_{pvs} \end{aligned}$$

14 $$\begin{aligned}&O_{ws} + I_{ws} \ge \sum _{p \in P} \sum _{v \in V^p} X_{pvws} - C, \ \forall w \in W&\end{aligned}$$

If any instances become infeasible before the end of the experiment’s planning horizon, we note the week that they become infeasible. This is further discussed in Section 6.

### Verification and validation

To validate our model, we obtained a data set from the health system that contained all obstetrics appointments that were scheduled at the clinic from March 1st, 2021–March 31st, 2022. In response to the COVID-19 pandemic, the health system began implementing a version of the tailored care paradigm, with reduced visit schedules for low-risk patients. Consequently, we compared the average utilization (i.e. number of appointments scheduled per week) and the average number of appointments per patient with those in the tailored care scenario of our model, under the protocol of scheduling all appointments up-front. Figure 4 shows the comparison in utilization between the model and historical data, where the shaded region indicates the range obtained from the model. Generally, the values obtained from historical data fall within this range, with a few exceptions. The historical data contains much more variation than the data obtained from the model, which is expected, as our model does not capture certain sources of variation that are inherent in reality. For example, our model does not capture variability due to changes in provider schedules, due to illness, vacation, or other factors. We were not able to obtain the provider schedules/number of appointments available per week for the time horizon considered in our data, making it difficult to draw clear conclusions about overbooking in our data. However, anecdotally, our clinical collaborators frequently cited overbooking as a common occurrence in their clinics, highlighting its importance in our experimental considerations. Additionally, our model over-estimates the number of high-risk patients within the system. This explains why the average number of appointments scheduled per week in the model (130.7) is higher than the average number of appointments scheduled per week in the historical data (123.8).

**Fig. 5:**
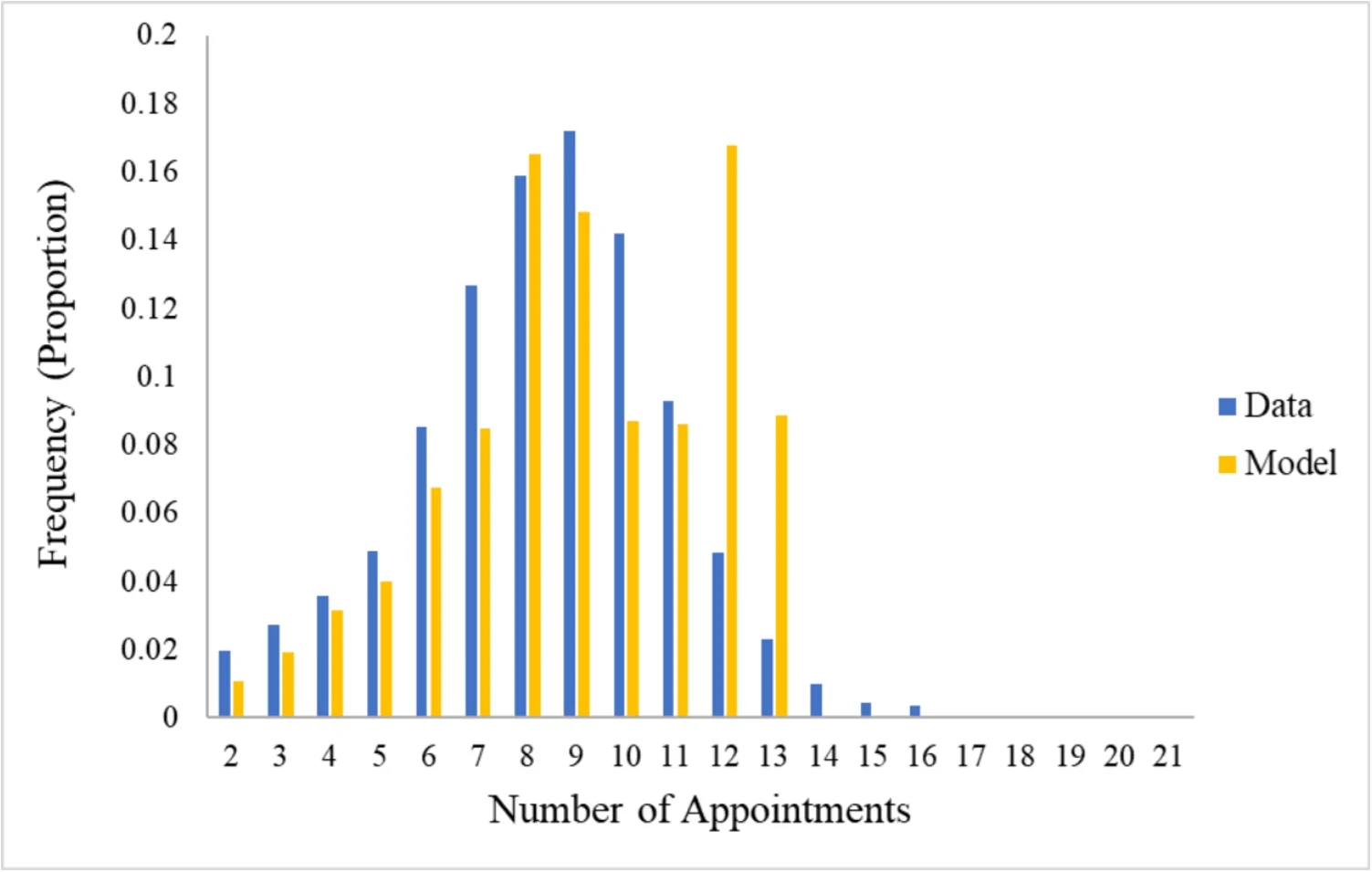
Distribution of the number of appointments per patient over the course of their pregnancy in the model and historical data

**Fig. 6:**
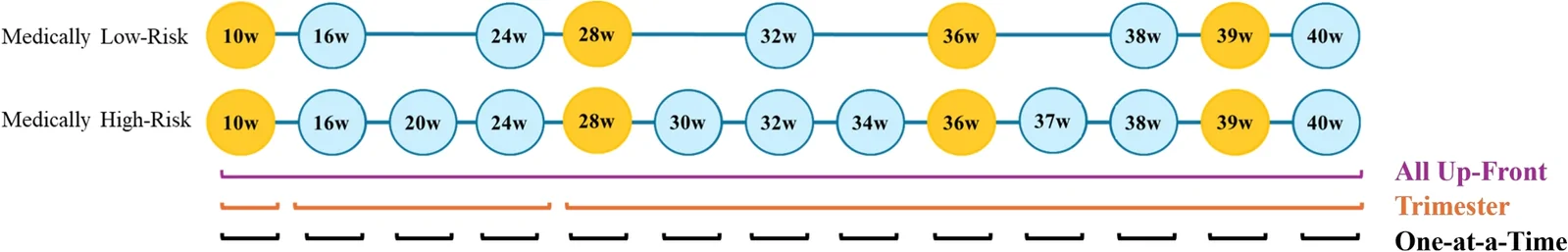
Scheduling protocols for prenatal care pathways

We also compared the average number of appointments per patient over the course of their pregnancy in the historical data and in our model (Fig. 5). The average number of appointments per patient in the model, across 170 replications, was 9.04 with a 95% confidence interval of [8.98,9.09]. The average number of appointments per patient in the historical data was 8.3 with a 95% confidence interval of [8.23,8.37]. As stated before, our model over-estimates the number of patients assigned to the high-risk pathway, meaning the average number of appointments per patient is expected to be higher in our model. Also note that in the model, we do not capture patients receiving appointments beyond their care pathway (e.g. a patient needs an extra appointment due to an unexpected health condition), as shown in the historical data, so it is impossible for a patient to receive more than 13 appointments in the model.

Due to the simplifying assumptions made in the model that leave out certain aspects of the real-world system, we supplemented the validation data analysis with expert opinion. We consulted with a physician from the clinic, who confirmed that the model’s outputs were within an acceptable range when compared to real-world clinic operations.

## Description of experiments

Our experiments are split into combinations of three levels:

1. Tailored vs. Non-Tailored: In the non-tailored scenario, all patients follow the 13-appointment pathway, while in the tailored scenario, medically low-risk patients follow the 9-appointment pathway and medically high-risk patients follow the 13-appointment pathway.
2. Appointment Scheduling Protocol: Patients can have all of their pathway appointments scheduled up-front when they initiate care, or they can be scheduled by trimester, or one at a time (Fig. 6).
3. Prediction Capability: We also compare the scheduling protocols to the “perfect knowledge" case, where the clinic has knowledge of all patient arrivals and their care pathways for the entirety of the planning horizon. In this case, we run the MILP once to schedule all patients for all of their pathway appointments up-front.

In addition to these levels, we also test the following cost schemes:

1. Status-Quo ($[c^T, c^O, c^Y]=[0.75, 0.25, 100]$: According to current practice, the clinic would rather overbook appointments than delay them. Additionally, appointments cannot be rescheduled.
2. Status-Quo with Rescheduling ($[c^T, c^O, c^Y]=[0.75,$ 0.25, 0.1]): Similar to the status-quo scheme, the clinic would rather overbook appointments than delay them. However, in this scheme, appointments can be rescheduled to their target weeks if capacity becomes available after the appointment has already been scheduled. For example, if a patient gives birth and their subsequent appointments are canceled, another patient who was previously delayed may be rescheduled to the newly opened appointment slot in their target week. A non-anchor appointment may also be rescheduled to a later week within its delay threshold to accommodate an incoming anchor appointment, if no open appointment slots exist within the anchor appointment’s delay threshold.

**Fig. 7:**
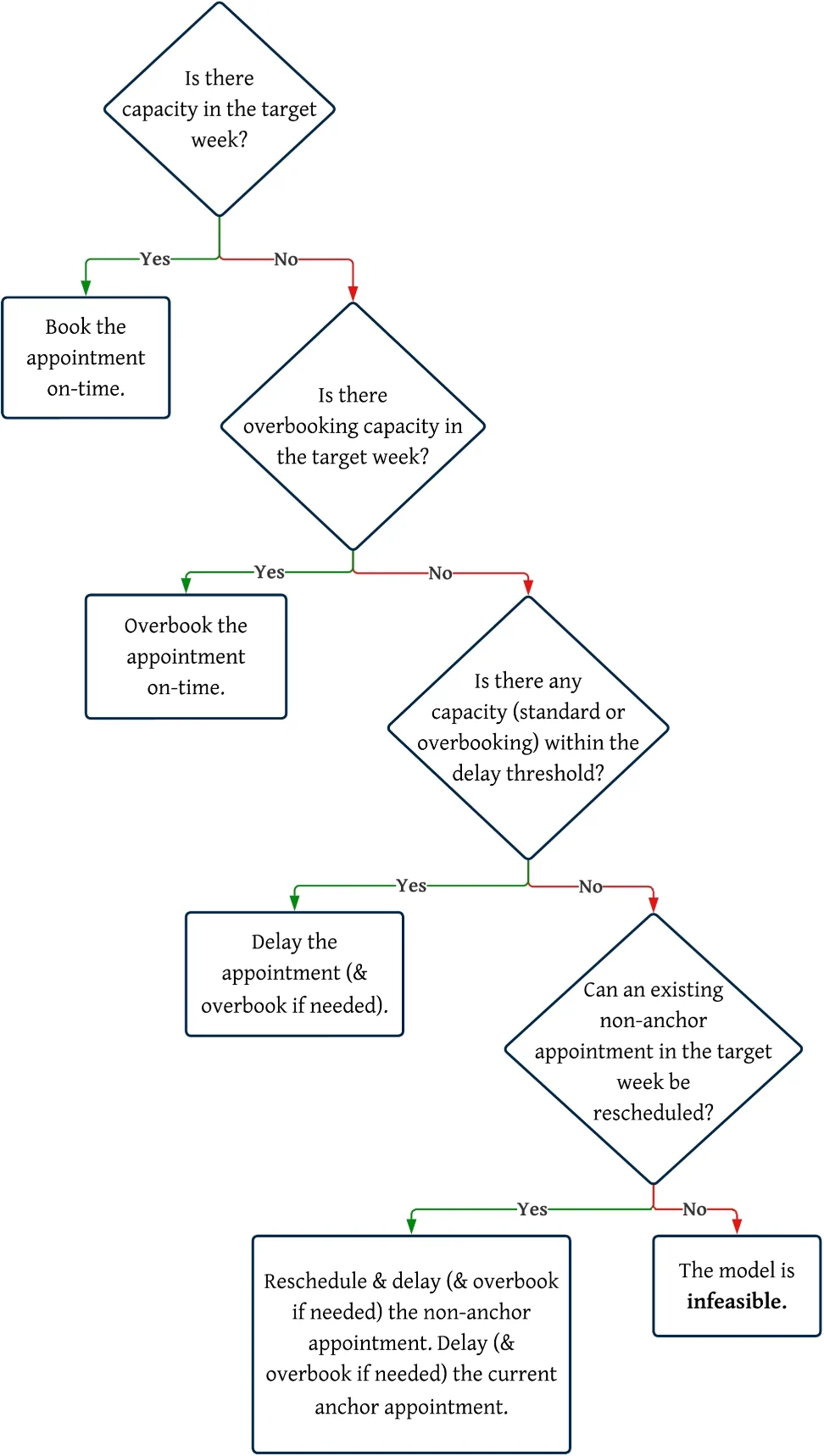
A decision flow for scheduling an anchor appointment under the $[c^T, c^O, c^Y]=[0.75, 0.25, 0.1]$ cost scheme. This logic dynamically adapts to alternative user-defined cost schemes

These specific cost schemes result in the following flow of decisions: if there is no capacity available in an appointment’s target week, the model first prefers to overbook the appointment in its target week. If there is no overbooking capacity available in the target week, the model then looks to see if there is capacity available (including overbooking capacity, if needed) within an appointment’s delay threshold. Given that rescheduling is allowed, if the appointment is an anchor appointment and there is no capacity available within the appointment’s delay threshold, the model tries to find a non-anchor appointment within the anchor appointment’s delay threshold that can be rescheduled to a later week. If none of these actions are possible, the model is infeasible, as the appointment cannot be scheduled at all. Figure 7 illustrates the decision flow for the “Status-Quo with Rescheduling" cost scheme. This flow can be changed depending on the cost scheme; for example, switching the costs of delaying and overbooking appointments in these cost-schemes would cause the model to choose to delay appointments before it considers overbooking.

We test different combinations of the scenarios and their settings and compare them against each other to draw insights about the best performing scheduling protocols. We also test these policies with different capacity levels at the clinic. The initial estimate of 156 appointment slots per week is based on the assumption that 50% of the clinic’s capacity (obstetrics and gynecology patients) is allocated to obstetrics. However, this number exceeds the average number of appointments scheduled per week in our model (130.7; estimated through the verification experiments in Section 4.3). Therefore, we also run experiments for the case where the clinic has 135 appointment slots, allowing us to compare policies when the clinic’s capacity is very close to the average weekly demand.

### Experiment settings

All experiments were conducted on a computer with the following specifications: Intel Core i9 computer with 64 GB RAM. Based on ad-hoc experiments using the status-quo, non-tailored scenario, with a half-width of 5%, 170 replications were determined to be sufficient. The warm-up period for the simulation model was 40 weeks, so each replication consisted of 92 weeks, to capture 52 weeks worth of data. The MILP was solved using IBM ILOG CPLEX optimization software, with the solver’s default settings.

**Fig. 8:**
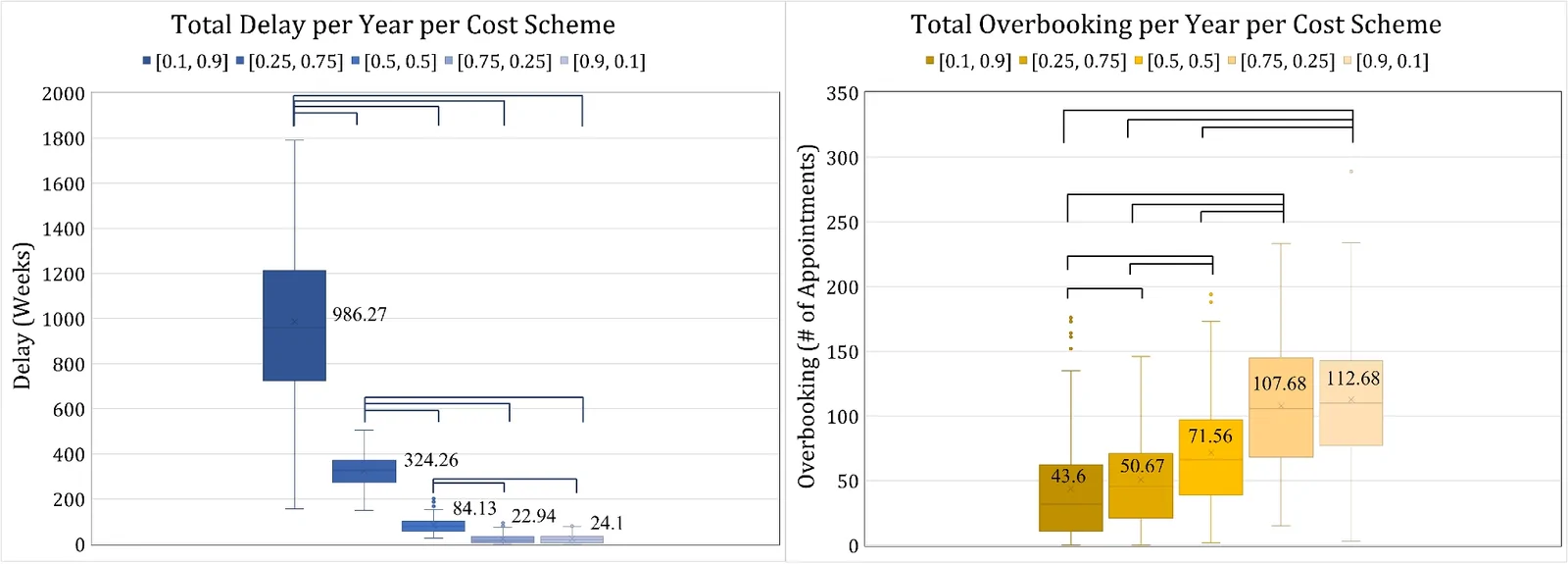
Delay and overbooking per year for varying cost schemes. The horizontal bars represent a statistically significant difference between the cost schemes, based on the wilcoxon rank-sum test

## Results

In this section, we summarize the key findings from our experiments. In Section 6.1, we present the results for the case where the clinic’s weekly capacity is 156, which we refer to as the excess capacity setting. In Section 6.1.1, we construct a Pareto front for varying provider preferences regarding delaying or overbooking appointments. In Section 6.1.2, we quantify the impacts of adopting the tailored care paradigm with respect to delaying, overbooking, and rescheduling appointments. While intuitively, adopting a policy with fewer appointments implies less delaying, overbooking, and rescheduling, these experiments highlight the magnitude and significance of these savings. In Section 6.1.3, we find that there is no significant difference in delaying, overbooking, or rescheduling between scheduling appointments by trimester or all up-front, but that scheduling appointments one at a time provides significant savings in all three metrics when compared to the other two scheduling protocols. We also find that scheduling appointments one at a time provides similar outcomes to the perfect knowledge case, implying that this scheduling protocol produces schedules that are very close to optimal.

**Fig. 9:**
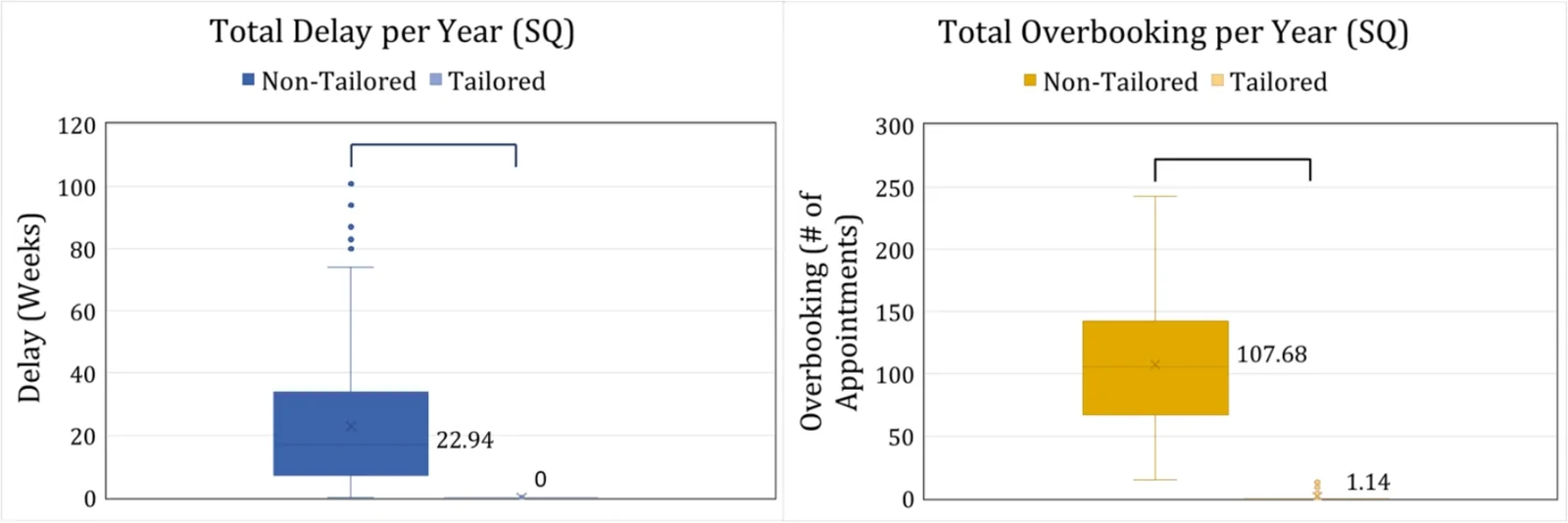
A comparison of delay and overbooking per year between the tailored and non-tailored care paradigms for the status-quo scenario. The horizontal bars represent a statistically significant difference between the paradigms, based on the wilcoxon rank-sum test

**Fig. 10:**
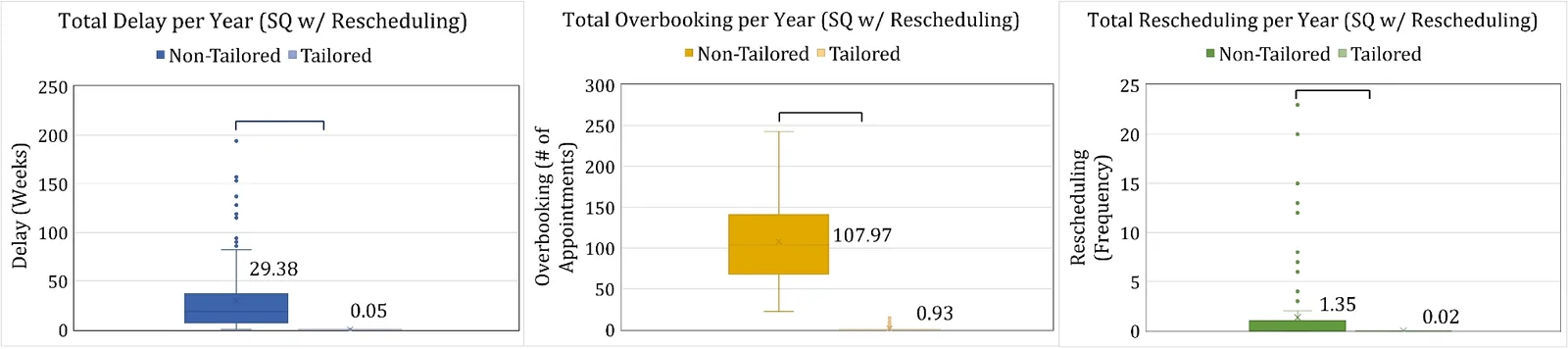
A comparison of delay, overbooking, and rescheduling per year between the tailored and non-tailored care paradigms for the status-quo with rescheduling scenario. The horizontal bars represent a statistically significant difference between the paradigms, based on the wilcoxon rank-sum test

In Section 6.2, we present the results for the case where the clinic’s weekly capacity is 135, or close to the weekly demand for appointments. We call this the limited capacity setting. In Section 6.2.1, we find that similar to the excess capacity setting, tailoring care significantly reduces the amount of delay, overbooking, and rescheduling in the system. In Section 6.2.2, we show that again, scheduling appointments one at a time provides outcomes that are most similar to the perfect knowledge case, but that all scheduling protocols perform significantly worse in terms of patient delay than the perfect knowledge case, highlighting the challenges and complexity of scheduling decisions with limited capacity.

Note that in all experiments, infeasibility occurs if the model overbooks an appointment beyond the overbooking limit. In reporting the final results, we include this overbooking in our metrics, but also record the first week in which the instance became infeasible (i.e., the first week in the horizon in which the number of appointments scheduled at the clinic exceeded the overbooking limit). Relatedly, any rescheduling of a non-anchor appointment to a later week, incurring delay and a rescheduling cost, only occurs if necessary to retain feasibility of the model.

### Excess capacity setting

While model run-times varied depending on the policy (e.g. the experiments where appointments were scheduled one at a time had a shorter run time than those where appointments were scheduled all up-front), the MILP took less than 15 seconds to run to optimality for all cases. Note that the MILP was solved 92 times for each replication (i.e., for each week in the experiment horizon), meaning each replication required less than 23 minutes of MILP solve time. The discrete event simulation model had a negligible run-time.

#### Comparison of cost schemes

In reality, providers may have different opinions about which actions they would rather take if a patient needs an appointment in a week that is already fully booked. To determine the impact of differing prioritization on the metrics, we tested the following cost schemes for the status-quo (i.e., providers can either delay an appointment or overbook it), non-tailored, schedule all appointments up-front scenario: [cost of one week of delay, cost of one overbooked appointment] = [0.1, 0.9], [0.25, 0.75], [0.5, 0.5], [0.75, 0.25], [0.9, 0.1]. We report the overall delay and overbooking for each cost scheme (Fig. 8). The horizontal bars represent a statistically significant difference between the cost schemes, based on the wilcoxon rank-sum test. The average delay and overbooking are also labeled.

As expected, as the cost of overbooking appointments decreases, the amount of overbooking in the system increases. The same is true for appointment delays. There is a significant difference in overbooking and delay between all cost schemes except between [0.75, 0.25] and [0.9, 0.1]. To capture the real-world preference of providers to overbook rather than delay patients, we use the [0.75, 0.25] cost scheme for all subsequent experiments.

#### Tailored vs. non-tailored care

To measure the impact of tailoring care, we compared the non-tailored care paradigm with the tailored care paradigm for the status-quo and status-quo with rescheduling scenarios. We used the schedule all up-front scheduling protocol for all experiments. Figure 9 shows the results for the status-quo scenario. Tailoring care significantly reduces the amount of overbooking and delay over the course of the year, entirely eliminating delay and reducing overbooking by approximately 98%.

Figure 10 shows the results for the status-quo with rescheduling scenario. As with the status-quo scenario, tailoring care significantly reduces the amount of overbooking and delay. Additionally, tailoring care significantly reduces the number of times appointments are rescheduled. Note that rescheduling did not happen often, even in the case of non-tailored care.

When comparing the status-quo and status-quo with rescheduling scenarios, there is no significant difference in delay and overbooking for both the tailored and non-tailored care paradigms. There also is not a significant difference in the capacity utilization; for both cost schemes, on average, the clinic filled 97% of their appointment slots per week in the non-tailored scenario, and 84% per week in the tailored scenario. Generally, the significant savings in all metrics is due to the reduction of appointment volume in the system when tailoring care – the fewer appointments there are in the system, the more easily the clinic can accommodate appointments in their target weeks.

**Fig. 11:**
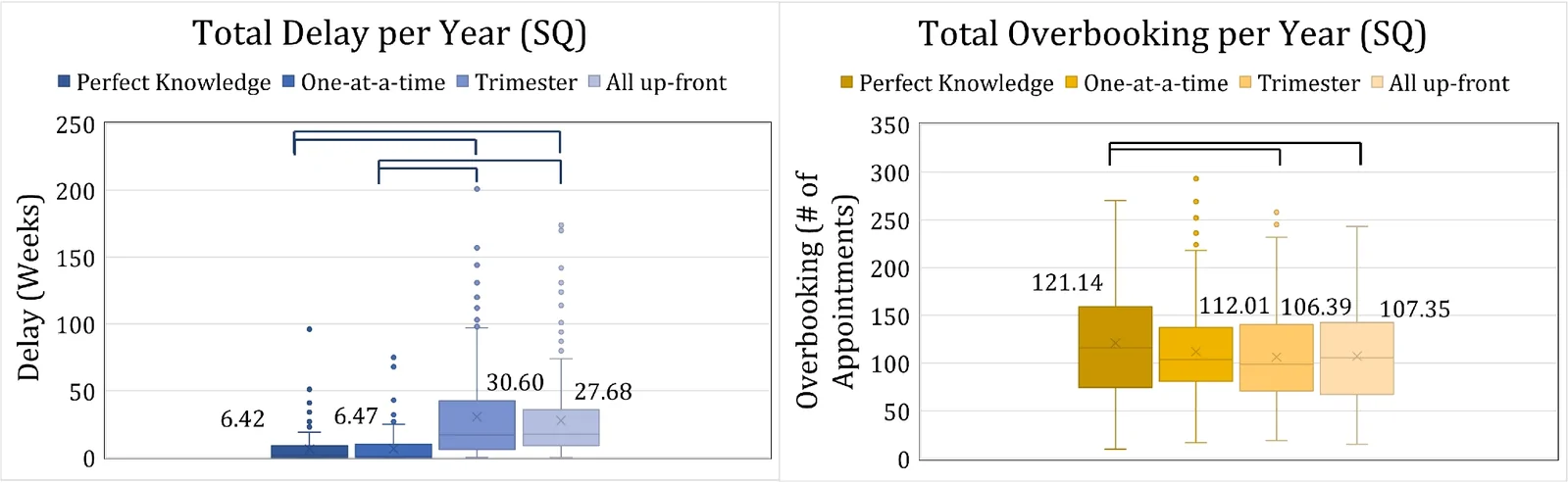
A comparison of delay and overbooking for varying scheduling protocols in the status-quo scenario. The horizontal bars represent a statistically significant difference between the protocols, based on the wilcoxon rank-sum test

**Fig. 12:**
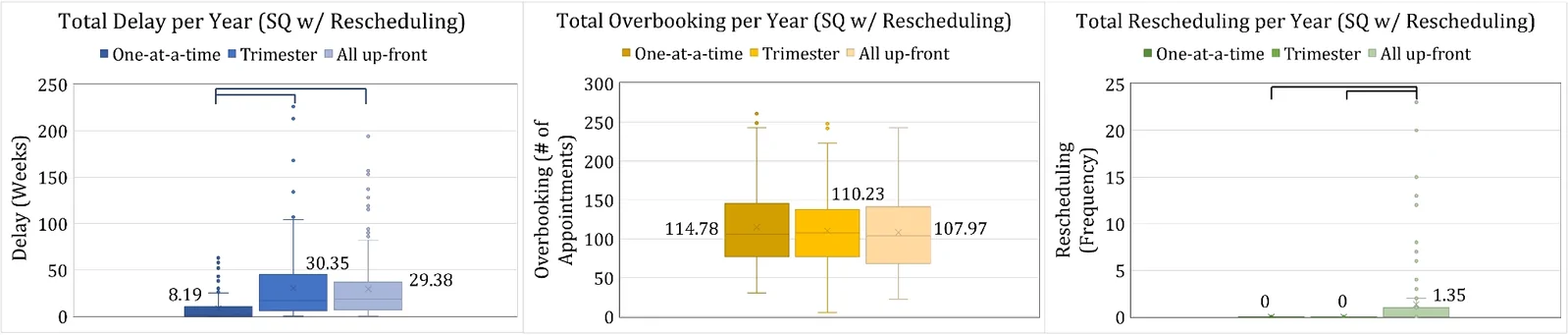
A comparison of delay, overbooking, and rescheduling for varying scheduling protocols in the status-quo with rescheduling scenario. The horizontal bars represent a statistically significant difference between the protocols, based on the wilcoxon rank-sum test

#### Appointment scheduling protocol and prediction capability

To measure the impact of different scheduling protocols, we compared all three protocols under the non-tailored scenario. We also included the results for the perfect knowledge case, where the clinic has knowledge of all patients and their pathways for the entire 92-week horizon. In the perfect knowledge case, we solved the MILP for scheduling patients once per replication, where all patients are scheduled for all of their appointments. We compared the perfect knowledge results with those of the three different scheduling protocols in the status-quo scenario (Fig. 11).

Scheduling by trimester or all up-front yields significantly higher delay than scheduling appointments one at a time. There is no significant difference in delay between scheduling by trimester or all up-front, due to the fact that the majority of the care pathway’s appointments are during the third trimester. There is also no significant difference in overbooking between all three scheduling protocols.

Additionally, there is a significant reduction in delay when the clinic has perfect future knowledge versus when the clinic schedules all up-front or by trimester with knowledge of only the current week’s arrivals. There is also a statistically significant increase in overbooking in the perfect knowledge case when compared to scheduling by trimester or all up-front, as more patients are able to be scheduled on-time. Conversely, there is no significant difference in delay or overbooking between the perfect knowledge case and when the clinic schedules appointments one at a time with knowledge of only the current week’s arrivals.

Figure 12 shows the results for the status-quo with rescheduling scenario. We do not include the perfect knowledge case, as rescheduling is not an option, since we only solve the MILP to construct the schedule once. Similar to the status-quo scenario, there is a significant decrease in delay when scheduling appointments one at a time compared to scheduling by trimester or all up-front. There is very little rescheduling in all scenarios, but there is still significantly more rescheduling when scheduling all up-front when compared to scheduling appointments by trimester or one at a time. There is no significant difference in overbooking. Note that rescheduling is most likely in the case of scheduling all up-front, as scheduling decisions are made further in advance of the actual occurrence of the appointment than in the trimester or one at a time cases. Likewise, rescheduling is least likely in the case of scheduling appointments one at a time.

**Table 3:** Feasibility information for experiments under the tight capacity setting

Scheduling Protocol	Cost Scheme	Perfect Information	% Infeasible	Average Infeasibility Week
One-at-a-Time	SQ	No	10%	57.4
Trimester	SQ	No	100%	43.6
All Up-Front	SQ	No	100%	36
All Up-Front	SQ	Yes	1%	66.5
One-at-a-Time	SQ w/ rescheduling	No	10%	63.4
Trimester	SQ w/ rescheduling	No	97%	71.9
All Up-Front	SQ w/ rescheduling	No	100%	43.6

**Fig. 13:**
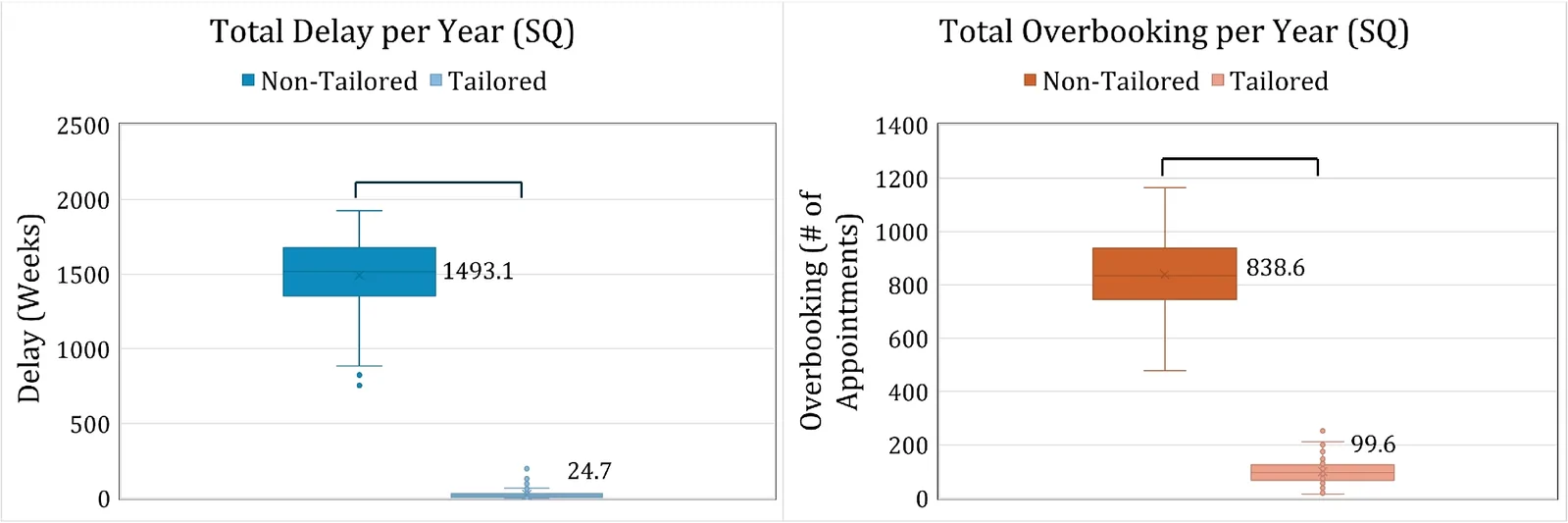
A comparison of delay and overbooking per year between the tailored and non-tailored care paradigms for the status-quo scenario, under the tight capacity setting. The horizontal bars represent a statistically significant difference between the cost schemes, based on the wilcoxon rank-sum test

As with the tailored versus non-tailored experiments, there is no significant difference in delay and overbooking between the status-quo and status-quo with rescheduling policies.

### Limited capacity setting

For this capacity setting, we ran the same experiments as those in Section 6.1 – comparing tailored vs. non-tailored care and scheduling protocols.

Similar to our experiments with the original capacity, we found that each replication took less than 23 minutes of MILP solve time, where each replication involved solving the MILP 92 times. However, some instances of the MILP required the usage of capacity beyond the clinic’s overbooking capacity, implying that the instance became infeasible before the end of the horizon. Note that here, we define infeasibility based on the “realized" capacity utilization, meaning that in the week that appointments actually occur, we capture the number of appointments that were overbooked. If in a certain week, the clinic’s schedule indicated appointments scheduled beyond the overbooking capacity, the instance was determined to be infeasible in that week. Table 3 summarizes the proportion of instances that became infeasible for each experiment, as well as the average week in the simulation horizon that the MILP model first became infeasible. Note that for the tailored care scenario, whether rescheduling was allowed or not, no instances were infeasible.

#### Tailored vs. non-tailored care

**Fig. 14:**
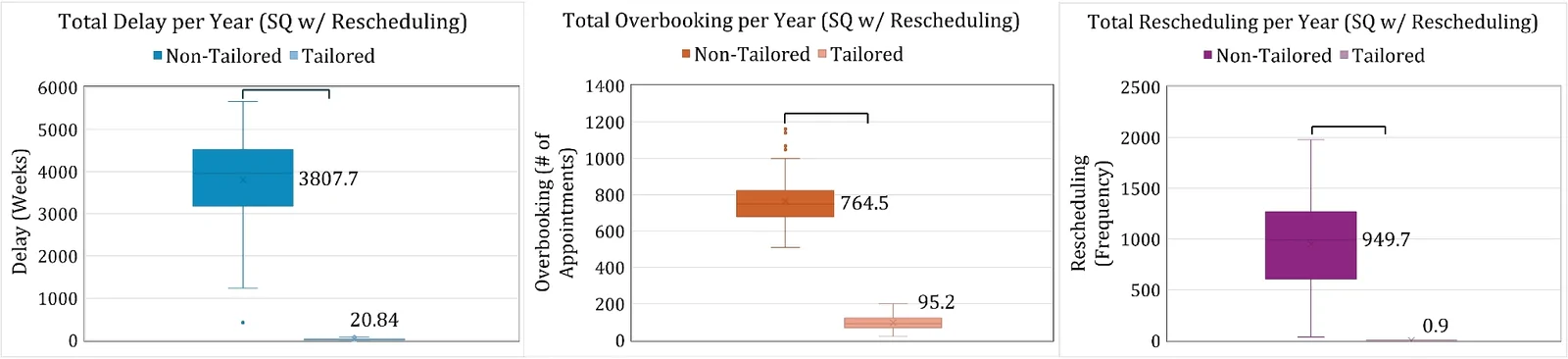
A comparison of delay and overbooking per year between the tailored and non-tailored care paradigms for the status-quo scenario with rescheduling, under the tight capacity setting. The horizontal bars represent a statistically significant difference between the cost schemes, based on the wilcoxon rank-sum test

As before, we compared the non-tailored care paradigm with the tailored-care paradigm, under the schedule all up-front scheduling protocol for both the status-quo and status-quo with rescheduling scenarios. Figure 13 shows the results for the status-quo scenario; again, tailoring care significantly reduces both delay and overbooking. On average, in the non-tailored paradigm, the clinic utilized 112% of their appointment slots each week, while in the tailored care paradigm, the clinic utilized 97% of their appointment slots. Figure 14 shows the results for the status-quo with rescheduling scenario. Again, tailoring care significantly reduced the amount of overbooking and delay. It also significantly reduced the amount of rescheduling, with almost no rescheduling occurring in the tailored care scenario. On average, the clinic utilized 111% of their appointment slots per week in the non-tailored paradigm, and 97% of their appointment slots in the tailored paradigm.

**Fig. 15:**
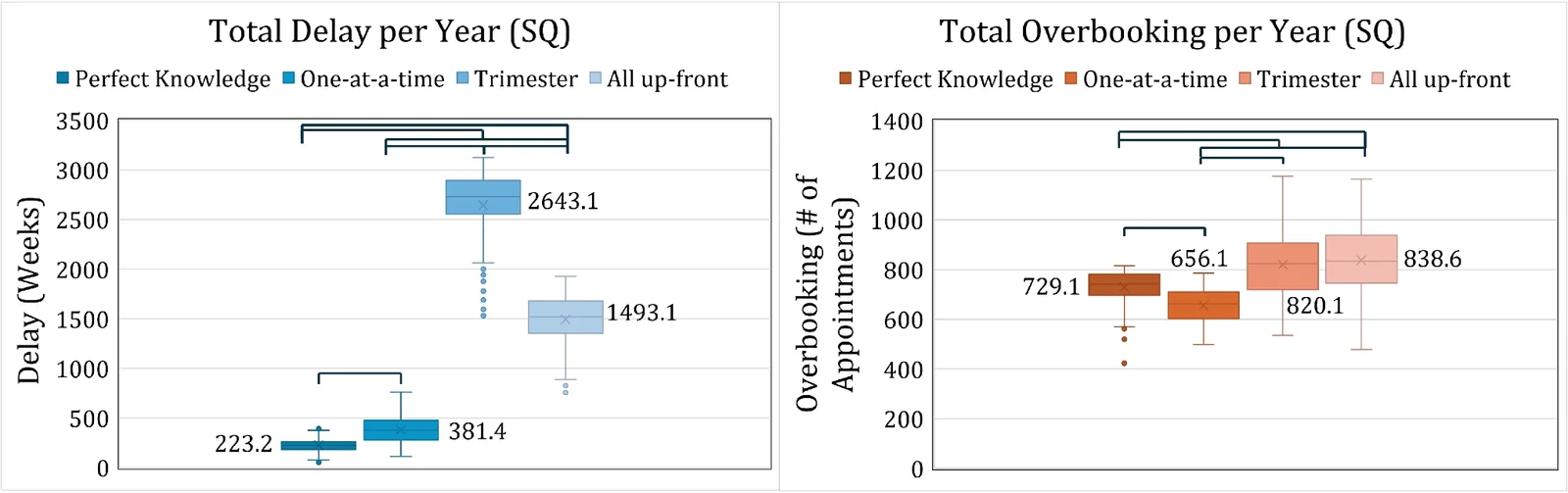
A comparison of delay and overbooking for varying scheduling protocols in the status-quo scenario, under the tight capacity setting. The horizontal bars represent a statistically significant difference between the protocols, based on the wilcoxon rank-sum test

When comparing the status-quo and status-quo with rescheduling scenarios, the status-quo scenario had significantly less delay, but significantly higher overbooking. This is due to the fact that in the status-quo with rescheduling scenario, existing non-anchor appointments were delayed to later weeks to accommodate incoming anchor appointments – note that non-anchor appointments have larger delay thresholds, meaning they can be delayed by more than one week. In the status-quo scenario, more overbooking occurred to maintain feasibility, as appointments could not be rescheduled.

When compared with the excess capacity setting, as expected, there is significantly higher delay, overbooking, and rescheduling (for the status-quo with rescheduling case), in all experiments. As the clinic’s capacity becomes more limited, it becomes more difficult to schedule appointments in their target weeks, leading to an increase in all metrics.

#### Appointment scheduling protocol and prediction capability

As in the excess capacity setting, we compared all three scheduling protocols under the non-tailored paradigm, including the perfect knowledge case for the status-quo scenario. Figure 15 shows these results. Scheduling by trimester or all up-front yields a higher delay than scheduling appointments one at a time, with scheduling by trimester far exceeding the other scheduling protocols. Significantly less overbooking occurs when scheduling appointments one at a time when compared with scheduling by trimester or all up-front, with no significant different in overbooking between the latter two protocols. When compared with the perfect knowledge case, scheduling appointments one at a time is the closest in terms of delay and overbooking, but the perfect knowledge case still has significantly less delay and significantly more overbooking than scheduling appointments one at a time. The perfect knowledge case yields significantly much less delay than when the clinic schedules by trimester or all up-front, and less overbooking as well.

**Fig. 16:**
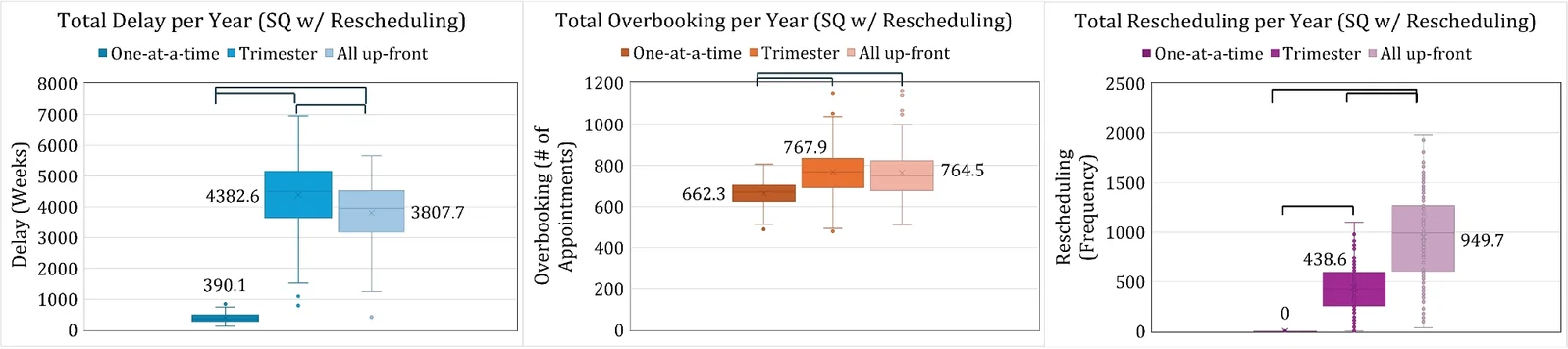
A comparison of delay, overbooking, and rescheduling for varying scheduling protocols in the status-quo with rescheduling scenario, under the limited capacity setting. The horizontal bars represent a statistically significant difference between the protocols, based on the wilcoxon rank-sum test

Figure 16 shows the results for the status-quo with rescheduling scenario. The findings are similar to the status-quo scenario, in that scheduling appointments one at a time produces significantly less delay and overbooking in the system when compared with the other protocols. Scheduling by trimester yields significantly higher delay than the rest of the protocols, but the difference is much less than that in the status-quo scenario. There is also significantly more rescheduling when scheduling appointments up-front, mainly due to appointments being in the schedule for longer.

When compared with the excess capacity setting, the results from the limited capacity setting show significantly higher delay, overbooking, and rescheduling (for the status-quo with rescheduling scenario) across all experiments. As in the excess capacity setting, scheduling appointments one at a time yields the closest results to the perfect information case.

## Discussion

Our framework proposes a discrete event simulation model that captures patient heterogeneity, with an MILP embedded within the model that optimally schedules patients for their prenatal care appointments. To quantify the quality of the constructed schedules, we defined three metrics: patient delay, overbooking, and rescheduling (if applicable). We considered two different cost schemes, reflecting the clinic’s prioritization of objectives: 1) status-quo, where the clinic would rather overbook than delay an appointment, with no rescheduling allowed and 2) status-quo with rescheduling, where the clinic would rather overbook than delay an appointment, but allows rescheduling in certain cases. We used this model to compare scheduling protocols (scheduling appointments up-front, by trimester, or one at a time) and to compare prenatal care paradigms (tailored or non-tailored care).

### Excess capacity setting

As expected, tailoring care significantly reduces the amount of delay and overbooking in the system over the course of the year, for both cost schemes, almost entirely eliminating delay. For the non-tailored care paradigm, of the patients that were delayed, 84% had a cumulative delay in care of one week during the entire gestational period, 10% were delayed by two weeks, 4% were delayed by three weeks, and the rest were delayed by four to six weeks over the course of the entire pregnancy. Note that these delays are for the entire pregnancy and may be the result of delaying multiple appointments, especially for patients who had a cumulative delay of more than one week. For example, a delay of six weeks implies that multiple appointments were delayed by one to three weeks, depending on the gestational age at each appointment. Delays per appointment were constrained by clinically defined delay limits (see Table 2). For the status-quo with rescheduling cost scheme, tailoring care also greatly reduces rescheduling, as there are fewer appointments in the system, but rescheduling does not occur frequently in either paradigm. In the non-tailored paradigm, of the appointments that were rescheduled, 53% were non-anchor appointments that were moved to later weeks to accommodate an incoming anchor appointment that could not be scheduled within its delay threshold (i.e., not enough capacity available). 47% of the rescheduled appointments were appointments that were initially delayed, but later on an appointment slot opened up in their target weeks (due to another patient giving birth and having their subsequent appointments canceled), causing the appointment to be rescheduled to its target week.

When comparing scheduling protocols, there is no significant difference in overbooking between the protocols, due to the patient volume and appointment demand being the same across all of them. Note that overbooking is slightly higher when scheduling appointments one at a time, implying that appointments are scheduled on-time more frequently, but the difference is not statistically significant. The protocols do differ significantly in terms of patient delay, however. Scheduling appointments one at a time yields the lowest amount of delay by a large margin (for both the status-quo and status-quo with rescheduling cost schemes), due to two main factors. First, at any given point in time, the number of appointments in the schedule is less than the number of appointments when scheduling by trimester or all up-front, meaning the likelihood that an appointment can be accommodated in its target week is higher. Additionally, scheduling decisions are made as close as possible to the week that an appointment occurs, so the scheduler benefits from a more accurate knowledge of the true capacity utilization in an appointment’s target week than when scheduling by trimester or all up-front. In the latter two scheduling protocols, the scheduler makes scheduling decisions far in advance of the week that an appointment is set to occur, meaning they are missing information about future patient arrivals that may also need to be scheduled in an appointment’s target week, or information about an existing patient that may give birth in the future and free up an appointment slot in an appointment’s target week. This is exacerbated by our limited rescheduling window: if an appointment is delayed, but set to occur within the next month, it is locked in and cannot be rescheduled to an earlier week even if an appointment slot opens up in its target week. In the status-quo with rescheduling cost scheme, there is very little rescheduling in all of the scheduling protocols (less than two times, on average), but scheduling all appointments up-front results in significantly more rescheduling, simply because appointments are in the schedule for a longer amount of time, and therefore have a higher chance of being rescheduled.

When comparing the perfect knowledge case with all other scheduling protocols, having perfect knowledge of the future significantly reduces patient delay when compared to scheduling by trimester or all up-front with limited knowledge. Conversely, having perfect knowledge of the future significantly increases overbooking, meaning in order to minimize the amount of delay, overbooking must occur. This is due to the clinic having knowledge of cases when patients deliver before expected, freeing up capacity and allowing the clinic to overbook when they otherwise would not have had any overbooking capacity available, preventing delay. As mentioned previously, imperfect knowledge of the future can cause the scheduler to make poor decisions, highlighted by the greater delay seen in the trimester and all up-front protocols, especially in a context where appointments are not eligible for rescheduling within a certain time window. Perhaps most importantly, there is no significant difference between scheduling appointments one at a time and the perfect knowledge case, meaning scheduling appointments one at a time results in a schedule that has the least amount of delay and overbooking possible among all scheduling protocols.

### Reduced capacity setting

In the reduced capacity setting, the clinic finds it even more challenging to manage its scheduling in a way that retains feasibility. To fully understand the results from the reduced capacity setting, it is important to highlight the order in which the clinic makes decisions if it does not have capacity to accommodate an appointment in its target week. Given that the cost schemes favor overbooking over delaying an appointment, the model first checks if the appointment can be overbooked in its target week (i.e., the overbooking limit has not been exceeded). If it cannot be overbooked, the model delays the appointment to the earliest week within its delay threshold (this could also mean that the appointment is overbooked and delayed if all weeks in its delay threshold are at full capacity, but have not exceeded the overbooking limit). If there is no capacity available within an appointment’s delay threshold (i.e., the overbooking limit has been exceeded), the appointment is overbooked in its target week, and the instance is considered infeasible.

As in the excess capacity setting, tailoring care significantly reduces delay and overbooking in all experiments. All instances also remained feasible in the tailored care scenario, implying that the appointment volume was more manageable for the clinic. Of the patients that were delayed in the non-tailored scenario, on average, 17% had a cumulative delay in care of one week during the entire gestational period, 14% were delayed by two weeks, 33% were delayed by three to five weeks, and 37% were delayed by six or more weeks. Again, these delays are for the entire pregnancy and may be the result of delaying multiple appointments, especially in cases where the patient was delayed by more than one week during their pregnancy. Additionally, tailoring care almost completely eliminated rescheduling in the status-quo with rescheduling cost scheme. Of the appointments that were rescheduled in the non-tailored scenario, 24% were appointments being rescheduled to earlier weeks due to capacity freeing up in their target weeks, and 76% were existing non-anchor appointments being rescheduled to later weeks to accommodate incoming anchor appointments.

Interestingly, scheduling by trimester yields significantly higher delay in both cost schemes due to the sequence of decision-making; when scheduling appointments by trimester, there are fewer appointments in the schedule when scheduling decisions are being made, meaning the likelihood that the model can find an appointment slot within an appointment’s delay threshold, before the instance becomes infeasible, is higher. Conversely, when scheduling all appointments up-front, there are more appointments in the schedule at any given point, meaning it is more likely that the model cannot find any capacity within an appointment’s delay threshold and the instance becomes infeasible. While 100% of the instances eventually became infeasible for both scheduling protocols, having slightly fewer appointments in the schedule overall when decisions are made implies somewhat more flexibility in the options available. Note that ultimately, there is no significant difference in the realized capacity utilization (and consequently, overbooking), as most appointments occur during the third trimester, but that the main factor influencing the increased delay when scheduling by trimester is the timing of the decision-making.

There is no significant difference in overbooking when scheduling appointment by trimester or all up-front, due to most appointments occurring in the third trimester, but there is significantly less overbooking when scheduling appointments one at a time. Most notably, there is significantly less overbooking when scheduling appointments one at a time when compared with the perfect knowledge scenario. This is due to the lack of knowledge about a patients’ delivery week – when scheduling appointments one at a time, due to limited capacity, the model may not be able to overbook an appointment in its target week, and instead chooses to delay it. If a patient delivers before this appointment occurs, the appointment is never realized, and is removed from the realized delay and/or overbooking metrics. However, when the model has knowledge of patient’s delivery dates, it is able to make better scheduling decisions, and no appointments are skipped.

When comparing cost schemes (i.e., status-quo vs. status-quo with rescheduling), there is no significant difference in delay and overbooking when scheduling appointments one at a time. However, there is significantly higher delay when rescheduling is allowed, and significantly less overbooking. This is due to the nature of the rescheduling – most rescheduled appointments were non-anchor appointments that were delayed to later weeks to allow incoming anchor appointments to be scheduled. There is slightly less overbooking in this case, as the model exceeded the overbooking limit less often.

In contrast to the excess capacity setting, scheduling appointments one at a time is significantly different than the perfect knowledge scenario; there is higher delay and less overbooking. However, scheduling appointments one at a time is still the best performing scheduling protocol among the three tested.

## Conclusions

In response to a growing need for restructured prenatal care, prenatal care experts have developed a new prenatal care paradigm, which moves away from the traditional “one-size-fits-all” model to one where patients receive tailored care based on their medical risk factors. As part of the implementation plan, prenatal care stakeholders are interested in the operational impacts of adopting new policies. Compared to previous literature related to this shifting prenatal care paradigm, our study offers a few novel contributions:

- Methodologically, our model embeds an MILP within a discrete-event simulation model, which allows us to easily model subtle differences in preferences for varying scheduling policies, namely the trade-offs between delaying, overbooking and rescheduling appointments. In our study, we propose two cost schemes, quantify the system’s behavior under these cost schemes, and show that tailoring care significantly reduces the amount of patient delay and overbooking in the system, improving the quality of care for patients while not burdening clinics. As demonstrated in Fig. 8, users can define their own cost schemes based on their specific preferences, which is especially relevant in health systems with varying provider preferences; for example, one provider may strongly prefer to overbook their patients, while others may be open to delaying or rescheduling. This model offers a simple way to model these dynamics, which would be significantly more complicated with a standalone discrete-event simulation model.
- Additionally, our study answers new, clinically relevant, questions about scheduling policies. Specifically, we model scheduling prenatal care appointments one at a time, by trimester, or all up-front, and find that scheduling appointments one at a time yields the best outcomes among the tested scheduling protocols, minimizing delay and overbooking, while providing physicians with the flexibility needed to dynamically adapt to patients’ evolving needs during pregnancy. These findings hold true even when the clinic has limited capacity.

These findings are particularly relevant in systems where capacity is limited, or the patient population consists of primarily high-risk patients. In a limited capacity setting, our results show that rescheduling non-anchor appointments may be required to avoid overbooking past the system’s overbooking limit. Additionally, scheduling appointments one at a time yields significantly less patient delay, while also ensuring that appointments are not booked beyond the system’s overbooking threshold.

Similarly, in a setting where the patient population is largely high-risk, appointment volume increases, implying similar insights to the limited capacity setting regarding rescheduling non-anchor appointments and scheduling appointments one at a time. Additionally, while our model does not incorporate the possibility of patients requiring appointments beyond their prescribed care pathways, high-risk patients are likely to require additional appointments throughout their pregnancy to manage complex complications. Scheduling appointments one at a time in this case is critical, as it allows the system to dynamically adapt to their evolving needs.

As with all models, ours is not without limitations. While we made our best effort to include the most important factors, both from a clinical and systems perspective, our model is still missing some aspects of the real-world prenatal care system. For example, the possibility of patients requiring care beyond their pathways, appointment cancellations, and variations in physician schedules. Further study will be needed to determine how to incorporate these factors in future versions of the model. We also recognize that many of our inputs were based on empirical distributions, so the model and its findings are highly dependent on the data used to derive the inputs. Nevertheless, our model provides useful insights about the proposed policies for our specific health system and scope.

Future work will include additional aspects of real-world prenatal care. One factor is varying provider schedules – our model assumes that the clinic has the same number of appointment slots per week, but in reality this may vary due to varying physician schedules. Also, patients may need appointments beyond their defined prenatal care pathway; additional data collection and analysis will be needed to study the timing, causes, and frequency of this occurring. We may also include ultrasounds and antenatal testing, which are conducted outside of routine prenatal care visits. Capturing the utilization of the associated resources would help to paint a more complete picture of medical care utilization during pregnancy.

Future work will also include a more meaningful representation of cost coefficients in our MILP. Currently, the costs associated with delaying, overbooking, and rescheduling are arbitrary weights, however, it may be more useful to calculate accurate costs, whether financial or health-related, from both the health system and patients’ perspectives, for each of the actions.

Future work might also include an analysis focused on how our policy recommendations change depending on patient and clinic-specific factors (e.g., patient volume, patient attributes, clinic characteristics, etc.). This analysis would help provide insight into how our recommendations would change when applied to a clinic different than our own.

Given the importance of social support in prenatal care, future work might also incorporate social support resources in the model. These services are often offered outside of routine prenatal care appointments through community organizations, public agencies, or other institutions. Further data collection and data analysis would be needed to determine the major organizations that provide these services to our patient population, as well as their specific capacity and utilization information. By extending the model to include social services, we could provide a more complete analysis of prenatal care systems while also drawing insights about how best to allocate and utilize these services.

As this model is further developed to incorporate more real-world behavior, we believe that it can also be developed into a clinic-facing tool to help guide capacity planning and scheduling. This could be in the form of a tool to be used by schedulers at the clinic level to help guide scheduling of patient appointments on a weekly or even daily basis, or at a higher level to help the clinic or health system with long-term capacity planning.
